# Seismic anisotropy prediction using ML methods: A case study on an offshore carbonate oilfield

**DOI:** 10.1371/journal.pone.0311561

**Published:** 2025-01-07

**Authors:** Guibin Zhao, Fateh Bouchaala, Mohamed S. Jouini, Umair Bin Waheed

**Affiliations:** 1 Earth Sciences Department, Khalifa University of Sciences and Technology, Abu Dhabi, UAE; 2 Mathematics Department, Khalifa University of Sciences and Technology, Abu Dhabi, UAE; 3 Geosciences Department, King Fahd University of Petroleum and Minerals (KFUPM), Dhahran, KSA; China University of Mining and Technology, CHINA

## Abstract

Estimating seismic anisotropy parameters, such as Thomson’s parameters, is crucial for investigating fractured and finely layered geological media. However, many inversion methods rely on complex physical models with initial assumptions, leading to non-reproducible estimates and subjective fracture interpretation. To address these limitations, this study utilizes machine learning methods: support vector regression, extreme gradient boost, multi-layer perceptron, and a convolutional neural network. The abundance of seismic features leads to many feature combinations, making the training and testing of machine learning models challenging. Therefore, a workflow has been developed to systematically inspect seismic features and select the most appropriate one for anisotropy estimation with reasonable accuracy. Synthetic data were generated using an earth model and well data within a finite difference numerical program. After thoroughly investigating synthetic data, the amplitudes of direct and reflected waves in the time and frequency domains were selected as input features to train machine learning methods. Optimizing the machine learning hyperparameters allowed the training and testing procedures to be completed with high accuracy. Subsequently, the optimized machine learning methods were used to predict Thomsen’s parameters, ε and δ, of a shaley formation in the zone area. To validate the predictions, the ε and δ estimated at a well location were compared with those obtained using a physics-based model, resulting in the least relative errors ranging from 2.92% to 7.14%.

## 1. Introduction

Seismic anisotropy refers to the directional dependence of seismic attributes within geological media, such as velocity and amplitude. It arises due to the presence of aligned and oriented structures within the Earth’s subsurface, such as mineral crystals, fractures, or formation bedding. Therefore, estimating seismic anisotropy is crucial when investigating fractured and finely layered geological media. An anisotropic medium can be vertical transverse isotropic (VTI), where seismic velocity varies with the wave propagation angle relative to the vertical axis, common in horizontally layered sedimentary rocks. In a horizontal transverse isotropic (HTI) medium, velocity varies with the wave propagation angle relative to a horizontal axis, often due to vertical fractures or aligned cracks. In addition to the directional dependence of velocity, anisotropy can be noticed through shear wave splitting (SWS). This phenomenon generates fast and slow shear waves propagating parallel and perpendicular to fractures in the azimuthal direction.

Thomsen [1] introduced three anisotropic parameters ε, δ and γ, and expressed them as follows,

C33=ρVP20°C11=ρVP290°C55=ρVSH290°C66=ρVSV290°C13=(4ρVP245°−C11−C33−2C44)2−[C11−C33]2412−C44ε=C11−C332C33γ=C66−C442C44δ=(C13+C44)2−(C33−C44)22C33C33−C44 (1)

where the angles 0° and 90° represent directions parallel and perpendicular to the anisotropy symmetry axis, respectively, of the compressional velocity *V*_*P*_, as well as the horizontal and vertical shear velocities, while *ρ* is the mass density.

The parameters *ε* and *γ* describe the difference in P-wave and S-wave velocities, respectively, between wave propagation along the symmetry axis and wave propagation perpendicular to it, whereas *δ* describes the difference in P-wave velocity for angles near the symmetry axis. Thomson’s parameters have been used in applications such as velocity modeling, fracture studies, amplitudes versus offset (AVO) analysis, waveform inversion, and rock physics modeling. Amplitude variation with angle and azimuth (AVAZ) inversion has been widely used to estimate the subsurface anisotropic properties [e.g. 2, 3]. The AVAZ is a good technique to retrieve fracture orientations from 3D surface data; it is based on the physical concept stating that a fracture acts as a spring; it deforms more when the strike is parallel to the wave’s polarization than when it is perpendicular. Rüger [4] modelled this concept, by expressing the reflection coefficient in a horizontally transverse isotropic medium in terms of the incident angle and azimuth. The inversion of first arrival of travel time can also be utilized for estimating the anisotropy parameters, as demonstrated by Newrick, Lawton [5] and Liu, Liang [6]. They conducted inversions using the travel time of first arrivals from a walkway vertical seismic profiling (VSP) dataset. The two methods mentioned above focus on limited seismic features, such as reflection coefficients and travel time. This may result in the underutilization of seismic data, which contains information linked to anisotropy. Full-waveform inversion (FWI) is another method for investigating anisotropic media, in which the whole wavefield is used to invert the anisotropic parameters [7–9]. Therefore, the FWI method can explicitly incorporate physical and geological information into the inversion process to model seismic wave propagation in complex media. The success of FWI heavily depends on the quality of the initial model. A poor initial model can lead to convergence to local minima, resulting in inaccurate subsurface images. FWI is computationally intensive, requiring significant computational resources and time, especially for 3D models and large dataset. Several techniques based on the SWS concept have been developed to assess the anisotropy in geological media. Tsuji, Hino [10] succeeded in assessing seismic anisotropy using walkaround three-component (3C) VSP data with the aim of revealing the stress state within the Kumano Basin. Their approach involved processing the 3C VSP data to extract the main shear events and analyze shear-wave splitting. Diaz-Acosta, Bouchaala [11] succeed to remove the overburden effect on anisotropy estimate in reservoir zones, by incorporating a multicomponent velocity analysis based on SWS. Shear velocity is one of the most important rock physics parameters because its azimuthal variation can be used to assess properties of anisotropy sources, such as fracture orientation. Due to the difficulty in oilfield shear wave velocity estimates, scholars have developed innovative ML methods to estimate shear velocity from conventional log data. From 2017 to 2019, some scholars adopted the Least Square Support Vector Machine (LSSVM), Adaptive Neuro-Fuzzy System(ANFIS) and Multi-Layer Perceptron(MLP) that are combined with Particle Swarm Optimization(PSO) to estimate shear wave velocity from petrophysical log data [12–14]. After 2019, deep-learning-based methods gradually replaced traditional machine learning methods due to their better ability for feature extraction. Therefore, advanced deep-learning methods were proposed, such as Long–Short Term Memory (LSTM) and Recurrent Neural Network (RNN) to estimate shear velocity from log data [15–23].

In general, physics-based inversion methods have some limitations, such as capturing complex nonlinear relationships between seismic data and subsurface properties and their sensitivity to the choice of the initial model. Owing to their capacity for automatic feature learning and nonlinear mapping with minimal human intervention, machine learning (ML) methods have been extensively integrated into seismic inversion problems. The ML methods are rooted in data-driven techniques and do not depend on physical knowledge. However, ML studies have scarcely explored estimating seismic anisotropy parameters, particularly from non-zero offset seismic data such as surface seismic or walkaway VSP data. Nguyen‐Sy, To [24] utilized Artificial Neural Network (ANN), Extreme Gradient Boosting (XGB) and Support Vector Machine (SVM) methods to predict the elastic stiffness of a transverse anisotropic media. Scholars have utilized features such as porosity, density, compressional stress, pore pressure, and burial depth, derived from core samples, for both training and testing processes. In their study, Lee, Lumley [25] employed several supervised machine learning methods, namely Regression Trees, SVM, Ensembles of Trees, and ANN. These methods were employed to estimate anisotropic elastic parameters in a shale formation, utilizing porosity, clay volume, and kerogen volume calculated from conventional Gamma-ray, neutron porosity, and density logs as ML features. After using synthetic seismograms to train an ML model, Sabinin, Chichinina [26] demonstrated the feasibility of a 2D convolutional neural network (CNN) using full seismograms as features to estimate normal and tangential weakness of fractures (Δ_*N*_ and Δ_*T*_) and Thomsen’s parameters (*ε*, *δ*, *γ*) in a horizontal transverse isotropic (HTI) medium. Ding, Cui [27] developed a novel Multilayer Linear Calculator Neural Network (MLLC) method. They optimized this method with the particle swarm optimization (PSO) algorithm. The ability of the MLLC method for mapping relationships between seismic attributes (*V*_*p*_, *V*_*s*_, and *ρ*) and Thomsen’s parameters, was validated by one synthetic common depth point seismic gather.

**Fig 1:**
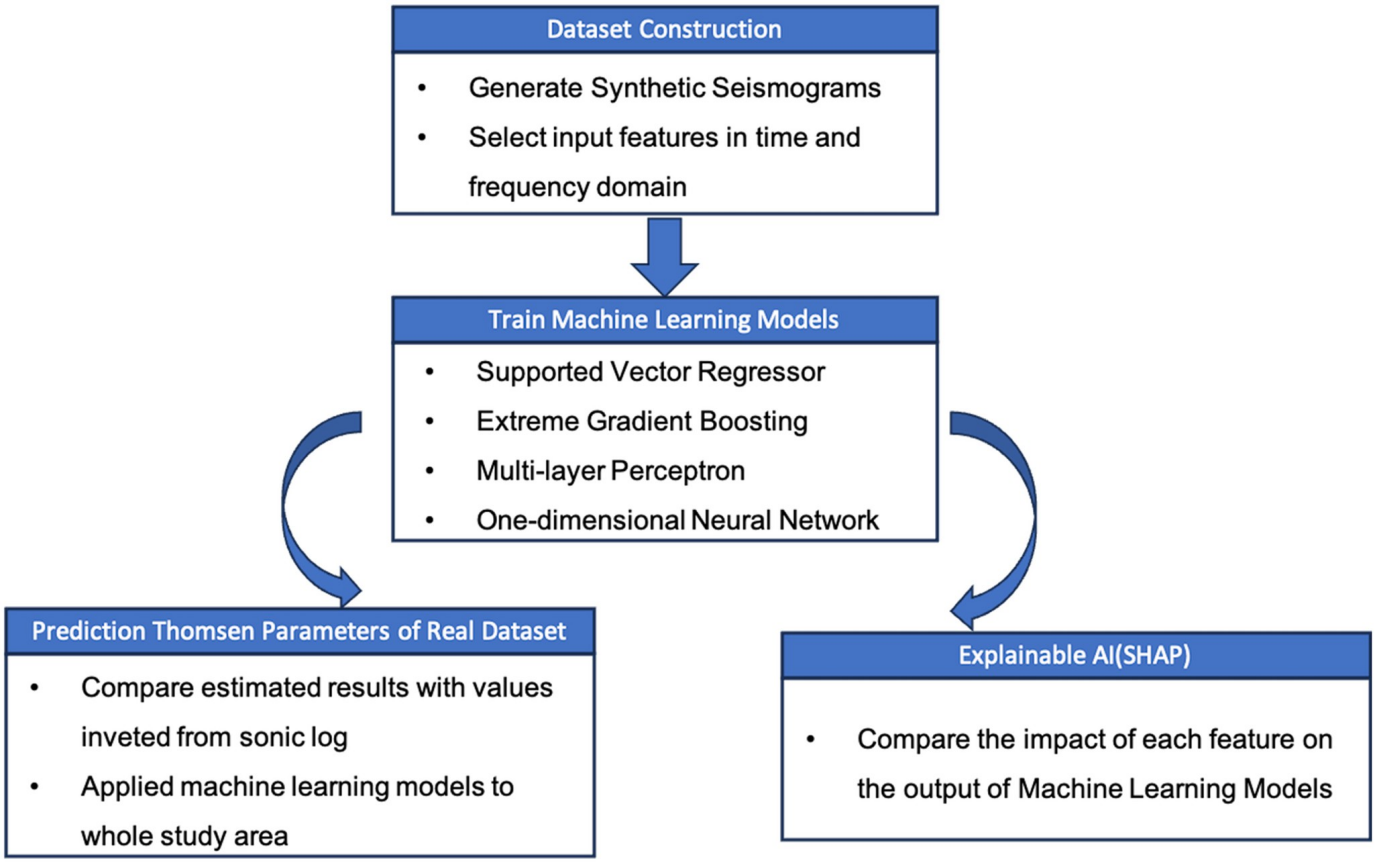
The machine learning workflow for estimating seismic anisotropy from non-zero offset synthetic and real data.

However, the ML studies are typically conducted at well locations or on synthetic non-zero offset seismic dataset. Scholars often grapple with more challenging issues when working with real seismic dataset than synthetic ones. This complexity arises from various factors, including noise, signal attenuation, and the intricate subsurface structure. Additionally, the abundance of features that can be extracted from seismic data results in many combinations for training and testing processes. Therefore, the primary objective of this case study is to develop a workflow (Fig 1) to evaluate the most effective ML methods and the most suitable seismic attributes for use as training and testing features, aiming to achieve a reliable estimate of anisotropy parameters. This workflow will serve as a baseline for future ML studies on seismic anisotropy.

ML models are trained as black-box models, making the rationale behind their decisions challenging to interpret. Therefore, Explainable Artificial Intelligence (XAI), which has become an active research field, is implemented in ML workflow to aid in understanding how these models make decisions. To date, there has been relatively little use of XAI within geophysics field. Noh, Kim [28] used the intrinsic XAI method, called prototype neural network, for the seismic facies classification, providing qualitative and quantitative explanation on how an entire model operates, whilst Birnie and Ravasi [29] leveraged self-supervised networks combined with XAI for coherent noise suppression. The explainable artificial intelligence approaches replace the need for any prior knowledge of noise itself.

This study tackles the challenge of estimating non-zero offset seismic data by using and optimizing the most popular ML methods. A large number of seismic features were inspected, before estimating Thomson’s parameters *ε* and *δ*, of a shale-dominated formation exhibiting a VTI anisotropy and overlying a carbonate reservoir. The Thomson’s parameter *γ* was not included, because our training only involved compressional data, which is unrelated to *γ*. Isolating shear waveforms from seismic data is challenging, especially in highly heterogeneous mediums such as the carbonates from which our data were obtained. Four machine learning methods were employed in this study, namely Support Vector Regression (SVR), Extreme Gradient Boosting (XGB), Multilayer Perceptron (MLP) and deep learning method (1D-CNN). After optimizing and training ML models with satisfied test accuracy, features extracted from the near offset (130 m offset) real VSP field shot gather were inputted into these ML models to estimate *ε* and *δ* of VTI anisotropy. Finally, we applied the trained ML models on field data recorded at different offsets spanning from 180 to 2000 m in order to identify the generalization ability of models.

## 2. Field dataset

The studied offshore oilfield is located in Abu Dhabi, United Arab Emirates (UAE), and it was shaped by two major compressional events in the Late Cretaceous and Tertiary [30]. The initial event occurred during the Late Cretaceous when Semail ophiolite and its corresponding northeast-southwest-oriented thrust sheets onto the Arabian continental margin [31, 32]. The subsequent compressional event occurred during the Neogene epoch when the Arabian Plate shifted northwestward, resulting in its collision with Central Iran along the Zagros orogenic front [32, 33].

During the deformation, burial, compaction, and doming periods due to Infracambrian salt had reconstructed the oilfield structure. Gently folded (1 to 3°), east-west elongated anticline structures dominate the primary trapping mechanism in the oilfield, and have created substantial hydrocarbon traps [34]. Extensional tectonics have influenced the field, resulting in a network of faults and fractures with dominant orientations of NW–SE to WNW–ESE [35, 36]. These features enhance the reservoir connectivity and permeability but complicate reservoir management.

The studied oilfield comprises ten formations, numbered sequentially from 1 at the top to 10 at the bottom. The reservoir is a part of the Lower Cretaceous group; it is made of porous, low-density, shallow-water, and grain-dominated stacked carbonate [37]. The reservoir is divided into three primary reservoir zones, which are separated by low-porosity, high-density, deep-water argillaceous carbonates commonly referred to as dense zones [38, 39]. Reservoir 1 is mainly made of pelagic micrite, whereas Reservoir 2 is dominated by poloidal micrite, and Reservoir 3 lithology mainly consists of dolomitic micrite [30, 37, 40]. Many low-impedance karsts and high-impedance channel deposits was revealed in the overburden formations [41].

**Fig 2:**
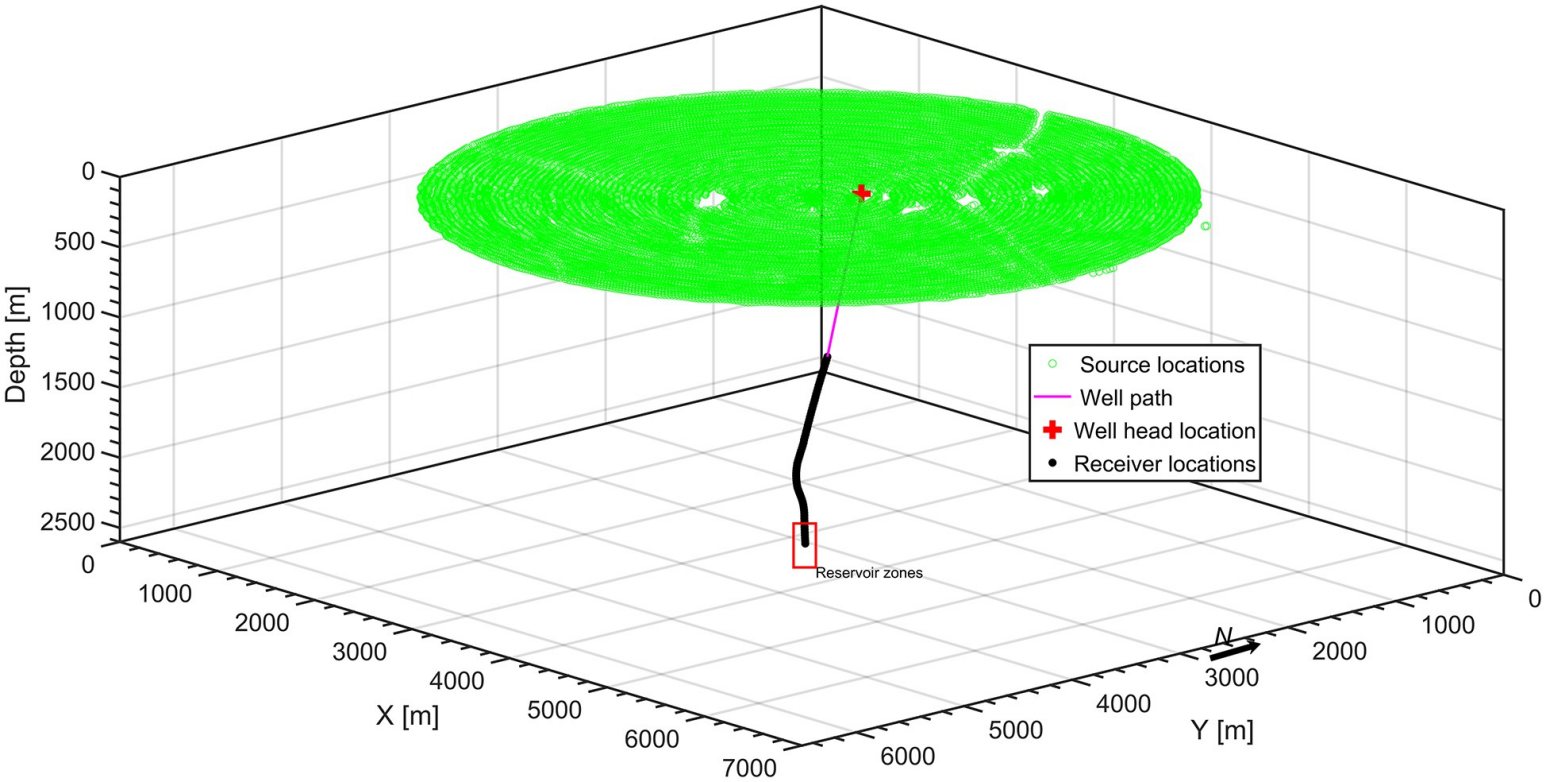
The spiral geometry of the 3D VSP survey was carried out in an oilfield in Abu Dhabi, UAE. The spiral spacing is 50 m, and the receiver spacing is 20 m. the red rectangular delineates the receivers located in the reservoir zone.

The 3D VSP survey comprises over 18000 shots spaced at intervals of 25 m along a spiral path, separated by 50 m (Fig 2). Shots were conducted with offsets from the wellhead ranging from 130 m to 3000 m, and data were recorded by seventy receivers positioned along a slightly deviated well with a vertical spacing of 20 m. The top twenty receivers were not considered into our dataset due to their low S/N ratio, while the 60^th^ receiver is dead without any signal record. Only compressional waves were utilized; therefore, only the Z component was considered in the dataset, as it primarily captures the compressional energy.

The Azimuthal travel time fitting technique [42] and shear wave splitting analysis, conducted in the reservoir zones and the overlying shaley formation number 10, acting as a cap rock, revealed that the horizontal transverse isotropy (HTI) is weak. There is a discernible HTI pattern, but it is weak, in the order of +/- 1ms. This means there is a large uncertainty in the fast P direction.

We assessed the VTI anisotropy in the reservoir zones and the two formations above by physically estimating Thomson’s parameters *ε* and *δ* at the wellhead location by following the steps below,

- Rotation of the 3C VSP data from local reference frame to geographical coordinate system [43], then the parametric wavefield separation technique “Wavesip” [44], was used to decompose the wavefield into downgoing P, downgoing Sv, upgoing P and Sv [43].
- Application of Slowness Polarization Inversion by using Monte Carlo global optimization method [45], to obtain a sequence of random samples from a probability distribution for which direct sampling is difficult. This sequence is used to approximate the distribution (i.e., to generate a histogram), and to compute an integral (such as an expected value). The confidence in the values is computed by the Monte Carlo histogram using 100000 models provided representations of the tilt axis symmetry and the ellipticity vs. anellipticity displays.

The Metropolis-Hastings algorithm implemented in Schlumberger’s anisotropy analysis toolbox is a Markov chain Monte Carlo (MCMC) method that obtains a sequence of random samples from a probability distribution for which direct sampling is difficult. This sequence is used to approximate the distribution (i.e., to generate a histogram) and to compute an integral (such as an expected value). The confidence in the values is computed by the Monte Carlo histogram using 100000 models provided representations of the tilt axis symmetry and the ellipticity vs. anellipticity displays.

**Fig 3:**
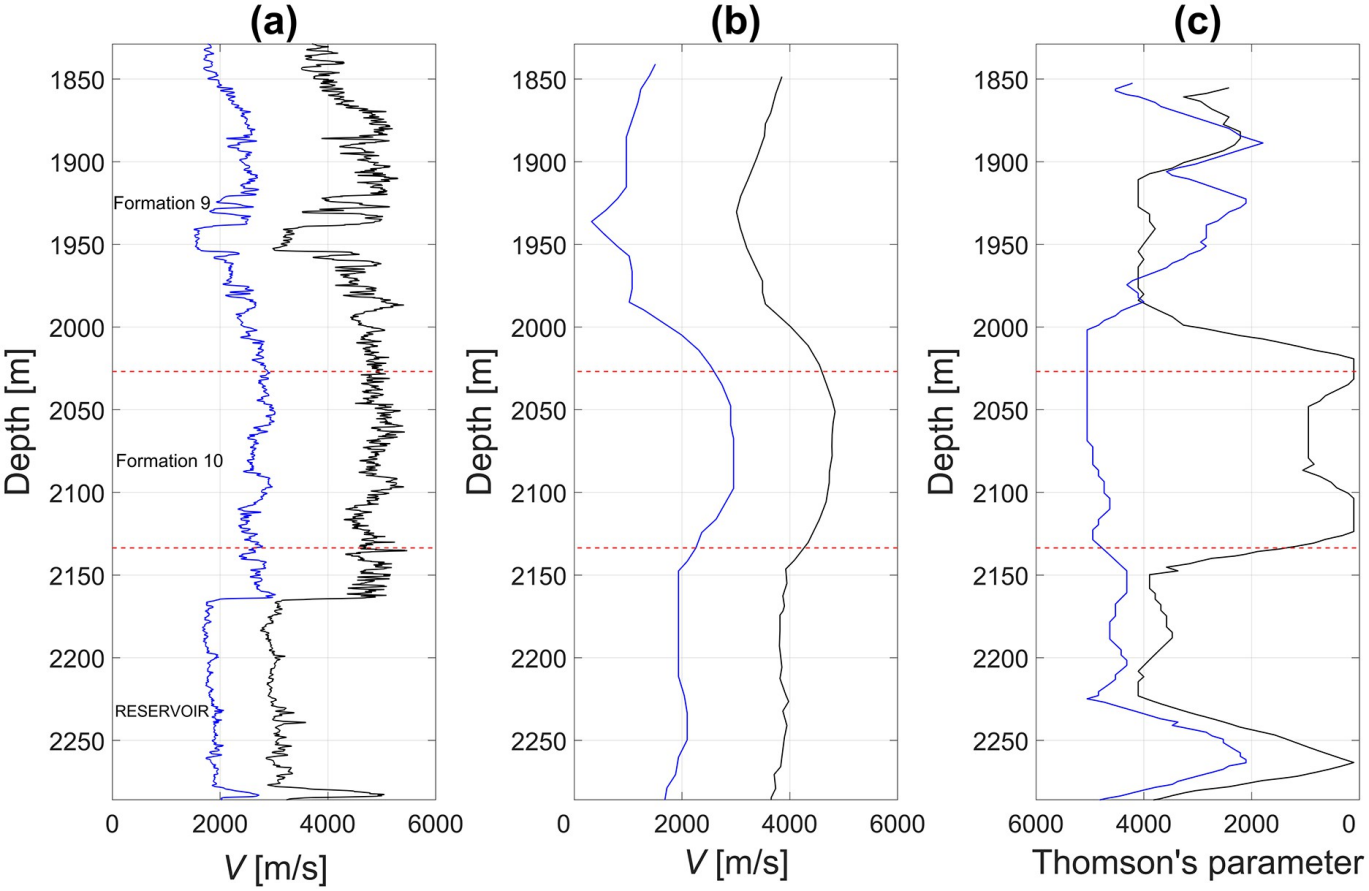
Compressional (black) and shear (blue) sonic logs (a) acquired in the field and (b) measured from polarization inversion by using Monte Carlo global optimization method (Leaney and Hornby 2007), and obtained ε (black) and δ (blue) anisotropy parameters. Dashed lines indicate formation tops.

The good correlation between the inverted *V*_*P*_ and *V*_*S*_ logs and the sonic logs (Fig 3a and 3b), demonstrates the accuracy of the inversion. The inverted *ε* and *δ* logs (Fig 3c), will be used as reference values for a comparison with the predicted ones by using machine learning models.

## 3. Machine learning methods

Several authors have applied advanced and novel machine-learning techniques to address seismic inversion problems. However, a major challenge lies in achieving a suitable fit between machine learning models and the dataset’s characteristics. Although more complex machine learning models, such as deep learning, are known for their high learning ability for complex patterns and thus good model accuracy, they also require massive amounts of data for training and computational time. In terms of our synthetic dataset, each set of input data is just composed of several extracted data points rather than sequential data or images. For our dataset, where complex patterns are not present, the data and computational efficiency of classical machine learning can be advantageous. Therefore, we selected three classical machine learning methods, namely, SVR, XGB, and MLP. Furthermore, we selected the deep learning method 1D CNN to increase the diversity of machine learning models. The success of ML critically depends on the setting of hyperparameters. We took into account the hyperparameter’s properties discussed below in the strategy of defining their values.

### 3.1 Support Vector Regression (SVR) method

**Fig 4:**
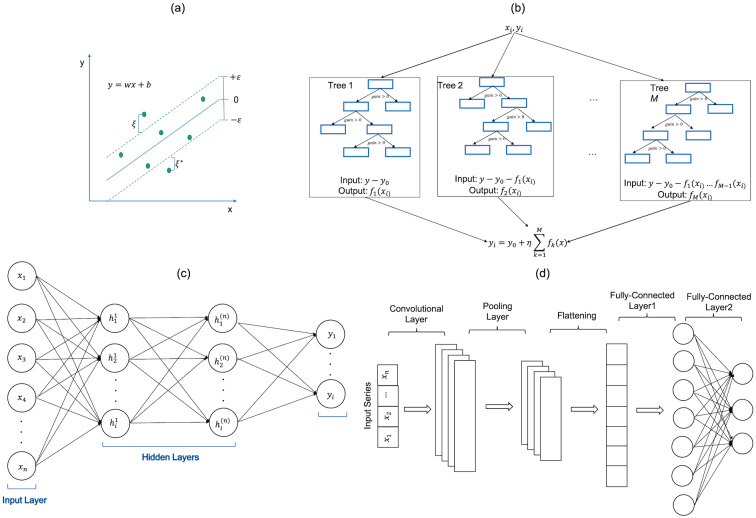
Illustrative diagram of (a) SVR method differentiating two classes by a hyperplane indicated by the blue line and bounded by two margins indicated by dashed lines, (b) XGB architecture where each rectangle represents a leaf belonging to a tree, (c) MLP layers composed of multiple nodes, and (d) 1D-CNN architecture.

The SVR method is built on the concept of SVM [46], which can handle non-linear data using kernel trick. The goal of SVM is to find a hyperplane in an N-dimensional space that clusters data points. Furthermore, the SVR method gives the flexibility to define how much error is acceptable and finds an appropriate line or hyperplane to fit the data. The hyperplane holds maximum training observations within the margin *ε*, as shown in Fig 4a. The optimization of the SVR method is achieved by minimizing a function of weights *w* while maximizing the margin. The SVR optimization problem can be formulated as follows:

$$\begin{array}{c} minimize\;\frac{1}{2}∥w{∥}^{2}\\ subject\;to\;\left\{\begin{array}{l} y_{i}-w_{i}-b\leq \varepsilon \\ w_{i}+b-y_{i}\leq \varepsilon \end{array}\right. \end{array}$$ (2)

To handle cases where some points may fall outside the margins, a technique called "soft margin" is employed. This is accomplished by introducing slack variables, denoted as *ξ*_*i*_, $\xi_{i}^{*}$, which allows for certain data points to violate the margin constraints. The modified optimization problem for soft margin SVR can be formulated as follows:

$$\begin{array}{c} minmize\left[\frac{1}{2}∥w{∥}^{2}+C\sum_{i=1}^{n}\left(\xi_{i}+\xi_{i}^{*}\right)\right]\\ subject\;to\;\left\{\begin{array}{l} y_{i}-w_{i}-b\leq \varepsilon +\xi_{i}\\ w_{i}+b-y_{i}\leq \varepsilon +\xi_{i}^{*} \end{array}\right. \end{array}$$ (3)

where *C* is a regularization parameter that controls the penalty assigned to the slack variables. In this study, we tune the value of *C* by finding a trade-off between minimizing the error and maximizing the margin. A large value of C requires a small margin to minimize the error, which causes a high risk of overfitting. By contrast, a smaller *C* allows a large margin.

To solve nonlinear problems using the SVR method, the kernel function transforms the training data to a higher dimensional feature space. One of the most used kernels is the Radial Basis Function (RBF), which transforms the space of features into an infinite space. The RBF kernel on two features *x*^(*i*)^ and *x*^(*j*)^ is defined as,

$$K\left(x^{\left(i\right)},x^{\left(j\right)}\right)=exp\left(-\overset{⌣}{\gamma }{\left\|x^{\left(i\right)}-x^{\left(j\right)}\right\|}^{2}\right)$$ (4)

where the term ${\left\|x^{\left(i\right)}\;-\;x^{\left(j\right)}\right\|}^{2}$ is the squared Euclidean distance between two features *x*^(*i*)^ and *x*^(*j*)^. Note that the variable $\overset{⌣}{\gamma }$ is a freely adjustable parameter that determines the level of influence exerted by two features on each other. For higher $\overset{⌣}{\gamma }$, the model will tightly capture the dataset’s shape, with more emphasis on fitting the training data, which may lead to overfitting on complex variable dataset. A compromise must be found during the tuning of $\overset{⌣}{\gamma }$ parameter, ensuring a good fit while avoiding the overfitting problem.

### 3.2 Extreme Gradient Boosting (XGB)

The XGB method is based on an advanced ML algorithm that belongs to the family of gradient-boosting methods. It combines gradient boosting with an ensemble of decision tree models to iteratively build a strong predictive model [47]. In this approach, the predicted results begin with a default value, typically the mean or median of the target variable. Subsequently, the predictions are refined by iteratively adding the output values from each tree (referred to as estimators) in the ensemble. Each tree attempts to correct the errors made by the prior one (Fig 4b), gradually improving the overall prediction accuracy as follows:

$$y_{i}=y_{i}^{0}+\eta \sum_{k=1}^{M}f_{k}\left(X_{i}\right)$$ (5)

where *y*_*i*_ is the predicted result of the *i*^*th*^ sample whose features vector is *X*_*i*_, $y_{i}^{0}$ is the initial value of the function. Noted that *M* refers to the total number of estimators (analogous to the number of boosting rounds), *η* is the learning rate that shrinks the feature weights to make the boosting process more conservative. More trees can improve the model’s accuracy but can also lead to overfitting. A lower value makes the learning process slower but can lead to better overall model performance due to more robust learning. However, it requires more estimators(trees) to maintain accuracy. The variable *f*_*k*_ is defined as the leave weight assigned to the k^th^ tree defined. The weights are determined by minimizing the objective function L defined as follows,

$$L=\sum_{i=1}^{n}l\left(y_{i},{\overset{ˇ}{y}}_{i}^{\left(k-1\right)}+\eta f_{k}\right)+\left(\frac{1}{2}\lambda O_{value}^{2}+\gamma T\right)$$ (6)

where *n* is the number of samples in the leave, *O*_*value*_ is the output value for the leave, *y*_*i*_ are the real values known from the training dataset, $\tilde{y_{i}}$ are the predicted values, *λ* is the regularization parameter to reduce the prediction sensitivity to individual observation, $\sum_{i=1}^{n}l\left(y_{i},{\overset{ˇ}{y}}_{i}^{\left(k-1\right)}+\eta f_{k}\right)$ is the loss function, while $\left(\frac{1}{2}\lambda O_{value}^{2}+\gamma T\right)$ is the regularization term and *T* is the number of *k*^th^ tree leaves. The number of leaves in a tree is often indirectly controlled by the maximum depth of a tree (max_depth) parameter, and other related hyperparameters. Increasing max_depth can model more complex patterns but may lead to an overfitting. Our strategy is to is to start with reasonable max_depth and increase it if the model underfits.

The loss function can be approximated using the Second Order Taylor Approximation technique as follows:

$$loss\approx \sum_{i=1}^{n}\left[L\left(y_{i},{\overset{ˇ}{y}}_{i}^{\left(k-1\right)}\right)+G_{i}f_{k}+\frac{1}{2}H_{i}f_{k}^{2}\right]$$ (7)

where $L\left(y_{i},{\overset{ˇ}{y}}_{i}^{\left(k-1\right)}\right)$ is the loss function at the previous step ${\overset{ˇ}{y}}_{i}^{\left(k-1\right)}$, *G*_*i*_ is the gradient of the *L* function, and *H*_*i*_ is the Hessian (second derivative of *L*). *L*(*y*_*i*_, *P*_*i*_) can be omitted, since they do not contain the output value. This results in a simplified objective function ℑ defined as:

$$ℑ=\sum_{i=1}^{n}\left[\left(G_{i}f_{k}+\frac{1}{2}(H_{i}+\lambda )f_{k}^{2}\right)\right]+\gamma T$$ (8)

The K^th^ tree is constructed by splitting the leaves starting from a single leaf. Such procedure is realised by maximizing the gain parameter defined as follows:

$$gain=\frac{1}{2}\left[\frac{G_{L}^{2}}{H_{L}+\lambda }+\frac{G_{R}^{2}}{H_{R}+\lambda }-\frac{{\left(G_{L}+G_{R}\right)}^{2}}{H_{L}+H_{R}+\lambda }\right]-\gamma$$ (9)

where *G*_*L*_ and *H*_*L*_ are the first and second gradients, respectively, of the loss function of overall samples in the leaf, *G*_*R*_ and *H*_*R*_ are associated to the right leaf after splitting. The splitting is accepted if the gain parameter is higher than 0.

### 3.3 Multilayer Perceptron (MLP) method

A Multi-Layer Perceptron [48] is a fully connected class of feedforward ANN consisting of an input layer, an output layer, and one or more hidden layers (Fig 4c). Each layer is composed of multiple nodes or neurons that perform computations of weights and biases based on the input data. Increasing layers and nodes helps to handle more complexity, thus enhancing learning ability. The neurons of input and hidden layers use activation functions to incorporate the non-linearity into the network, enabling it to learn complex patterns in the data. The predicted output *y* of each node is calculated as follows:

$$y=f\left(\sum_{j}w_{j}x_{j}+b\right)$$ (10)

where *x*_*j*_ is the input to the node *j* having a weight *w*_*j*_, *b* is the bias, *f* is the non-linear activation function and *y* is the output of the node.

The output of each node in a neural network is used as input for the nodes in the subsequent layer. This propagation of outputs through the network’s layers allows for the computation and transformation of the input data as follows:

$$h_{i}^{\left(n\right)}=f^{\left(n\right)}\left(\sum_{j}w_{ij}^{\left(n\right)}h_{j}^{\left(n-1\right)}+b_{i}^{\left(n\right)}\right)$$ (11)

where *f*^(*n*)^ represent the different activation functions at different layers, $h_{i}^{\left(n\right)}$ represents the weighted sum of inputs for the i-th node at the n^th^ hidden layer, *j* is the total number nodes the (n-1)^th^ hidden layer, and *y*_*i*_ is the weighted sum of inputs for the i^th^ output neuron.

The difference between the predicted and real outputs is computed using the loss function. The backpropagation process calculates the gradient of the loss function with respect to each weight and bias in the network. The optimizer takes the computed gradients and performs the weight updates during the optimization process. The choice of optimizer affects how quickly the neural network learns, how it avoids local minima, and how it converges to a solution. As it is commonly used, we choose to incorporate stochastic gradient descent (SGD), Adam (Adaptive Moment Estimation), and RMSprop optimizers.

### 3.4 One-dimensional Convolutional Neural Network (1D-CNN) method

The 1D-CNN method is a type of neural network commonly used for processing one-dimensional data, such as sequences or time series. It applies a set of filters (also known as kernels) to the input data. Each filter is a small window of weights that slides over the input sequence along one direction, calculating the dot product between the filter weights and the corresponding input values. This operation is called one-dimensional convolution. Larger kernels can capture broader features in the input sequence, which can be useful for detecting features that span multiple time steps. Smaller kernels are better for detecting more localized features. The kernel size choice can significantly affect the model’s performance and computational efficiency. Increasing the number of filters can allow the network to capture more complex features from the input data, potentially improving model accuracy. A CNN model typically consists of a sequence of different types of layers, including convolutional layers, pooling layers, and fully connected layers (Fig 4d). Convolution layer is the centerpiece of CNN, which can capture local patterns in a sequence by sliding the filter over the input. Pooling layers are used to reduce the size of the inputs and highlight the prominent features. However, excessive pooling might lead to the loss of important information. Fully connected layers, also known as dense layers, connect every neuron from the previous layer to every neuron in the next layer. Assuming a length of input *x* into 1D-convolution layer to be *n*, the size of kernel *h* to be *k* and the stride of sliding kernel to be *s*. The 1D-convolution can be defined as:

$$y\left(n\right)=\sum_{i=0}^{k}x\left(n+i+\left(s-1\right)\right)h\left(i\right)$$ (12)

### 3.5 Shapley Additive exPlanations (SHAP) model

Lundberg and Lee [49] proposed SHAP framework for explaining the predictions of machine learning models. The Shapley value comes from cooperative game theory and represents the average marginal contribution of a feature when all possible coalitions of features are considered. To compute the Shapley values, SHAP applies the concept of "coalitions" to the feature space. It considers all possible subsets of features and measures their impact on the model’s prediction. By considering all possible combinations, SHAP can capture interactions and dependencies between features. The Shapley value explanation is expressed as a method of additive feature attribution, defined as follows:

$$g\left(z^{\prime }\right)=\phi_{0}+\sum_{j=1}^{N}\phi_{j}z_{j}^{\prime }$$ (13)

where *g* stands for the explanation model, *z*′ ∈ {0, 1}^*N*^ is the coalition vector, *N* is the maximum coalition size and $\phi_{j}\in \mathbb{R}$ is the feature attribution for a feature *j*, called the Shapley value.

## 4. The workflow of ML anisotropy prediction

### 4.1 Synthetic dataset construction

To train the machine learning models, one thousand synthetic shot gathers were generated by varying Thomson’s parameters in a finite-difference wave propagation model in an anisotropic medium. The finite-difference model discretizes the wave equation in the space-time domain to approximate the propagation of seismic waves. Synthetic data were generated using a geological model consisting of eleven horizontal layers, with thicknesses defined from the well tops. The velocities and densities of these layers are determined as the averages of sonic and density logs in each respective formation. The dimensions of the background model are 500 m × 2500 m, and all layers are considered as Vertical Transverse Isotropic (VTI). Thomson’s parameters *ε* and *δ* were averaged in each formation, except in the target formation number 10, where the values of ε and δ are ranging from 0.1 to 0.2 and -0.1 to 0, respectively (Table 1). A point source emits a signal of a Ricker-shaped wavelet with a peak frequency of 30 Hz. The source is located on the earth’s free surface with coordinates x = 0 and z = 0. The shot-wellhead offset is 130 m, and the forty-nine receivers were placed in the well with 25 m vertical spacing.

**Table 1:** The 1–D earth model used as an input for simulating wave propagation in anisotropic media.

Layers	Thickness (m)	P-wave velocity (m/s)	S-wave velocity (m/s)	Density (g/cm^3^)	*ε*	*δ*
Formation 1	504	3300	1944	2300	0.0015	0.0010
Formation 2	847	3695	1944	2325	0.0224	0.0492
Formation 3	1303	4140	2177	2413	0.0490	0.0594
Formation 4	1584	4301	2249	2386	0.0616	0.0616
Formation 5	1815	3621	1815	2516	0.0385	0.0388
Formation 6	1917	4382	2209	2475	0.0417	0.0230
Formation 7	1951	4060	2022	2595	0.0329	0.0196
Formation 8	2153	4768	2596	2558	0.0209	0.0163
Formation 9	2179	3719	2185	2567	0.0103	0.0101
Formation 10	2289	3123	1886	2530	0.1–0.2	-0.1–0
RESERVOIR	2500	4199	2244	2395	0.0581	0.0082

**Fig 5:**
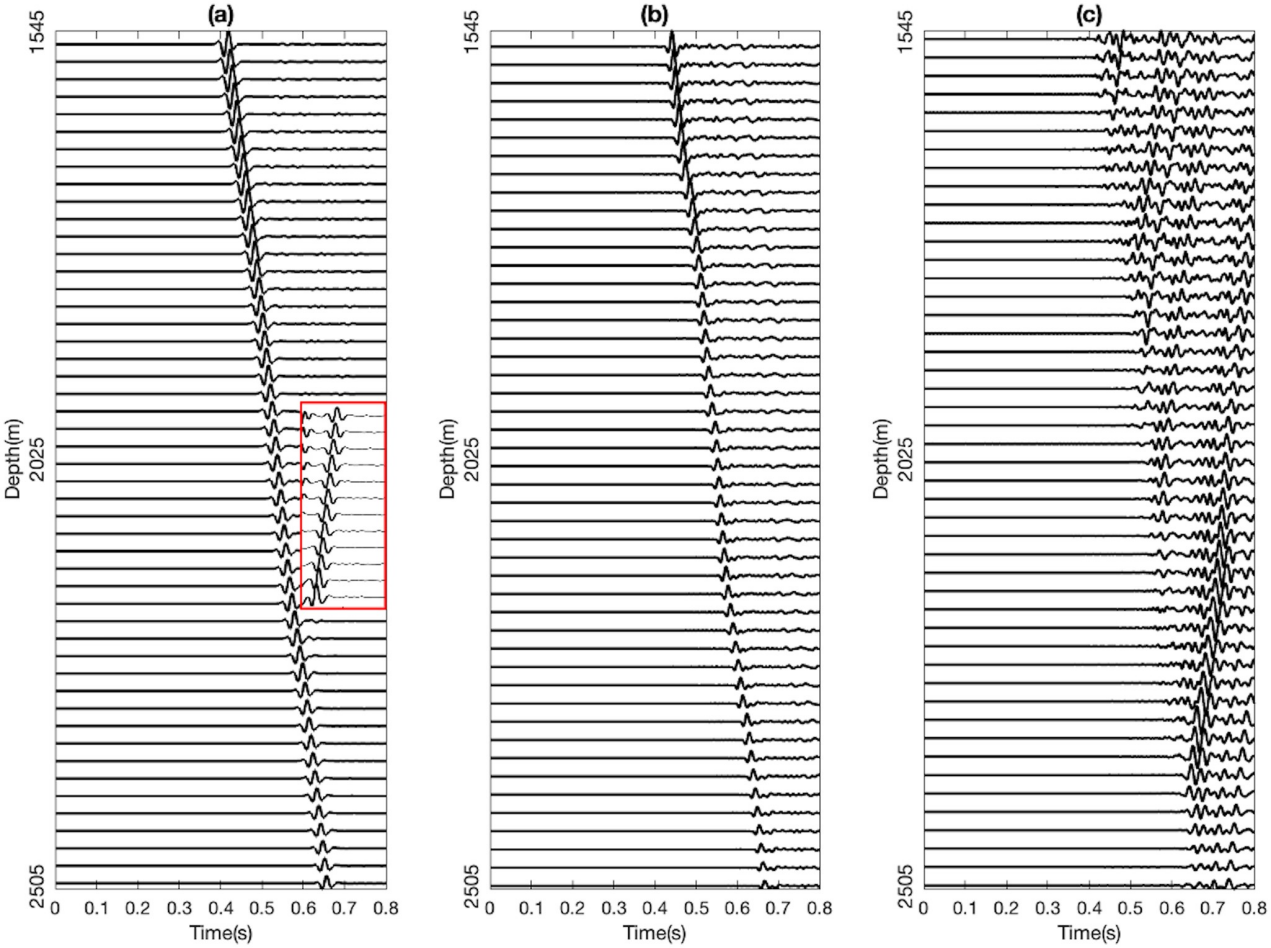
Synthetic shot gathers are generated in the (a) vertical direction using machine learning models. The reflected waves from the 22nd receiver to the 33rd receiver are zoomed, shown in the red square. (b) Downgoing waves and (c) upgoing of the field shot’s vertical component gathers.

The synthetic seismograms vary from each other because of changes in ε and δ values of the target layer number 10, randomly chosen within the mentioned intervals above, while all other layers remain unchanged across different models. These *ε* and *δ* values of each model act as the labels for the training dataset. In total, one thousand shot gathers were generated, with each shot gather comprising forty-nine traces of 0.8 s length, generated for each pair of *ε* and *δ* in the target formation. Both Ux and Uz components were generated, but only the Uz component (Fig 5a) was utilized in ML modelling.

### 4.2 Input features of training dataset

#### 4.2.1 Time domain features

**Fig 6:**
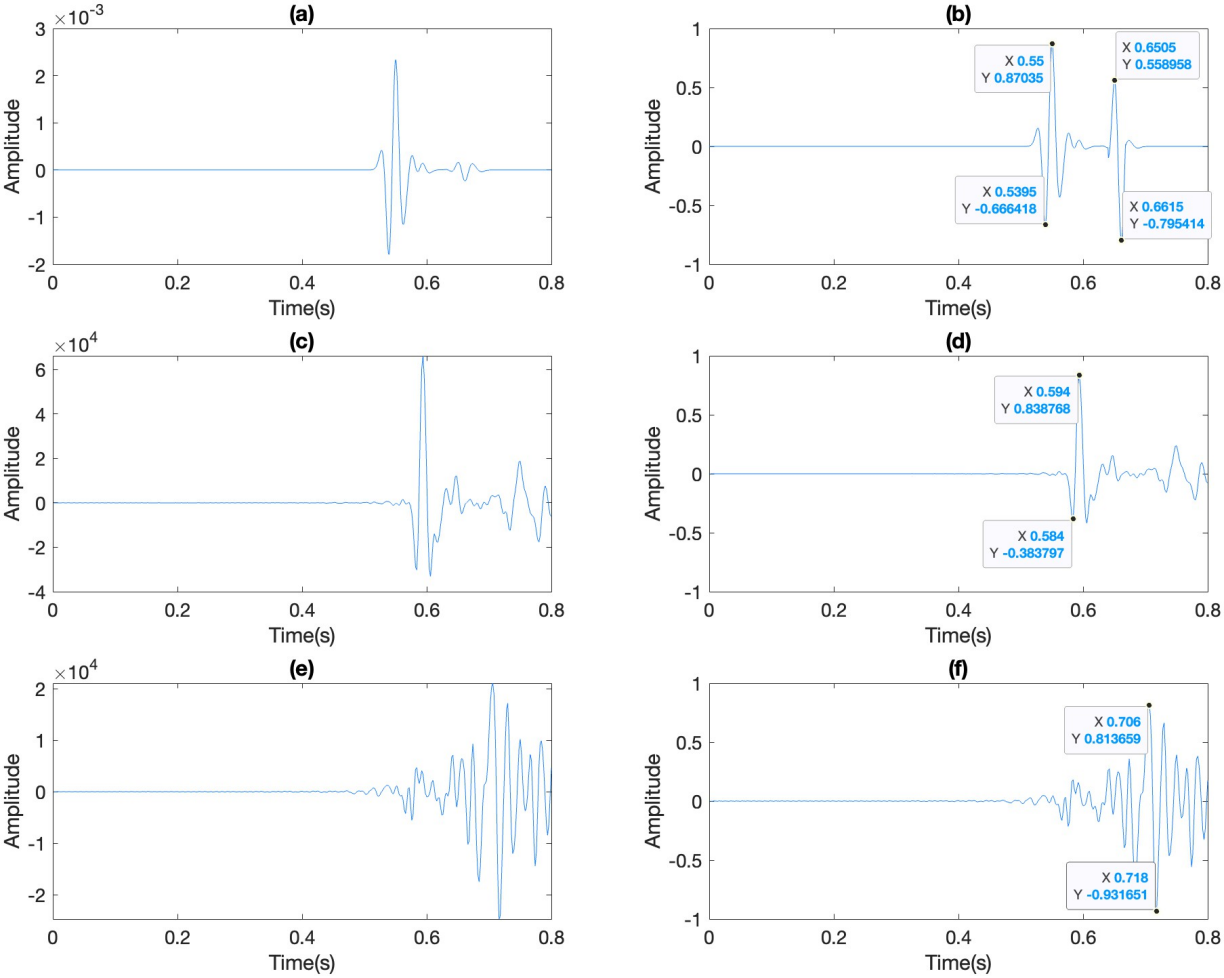
Seismic waveforms before (first column) and after (second column) normalization of (a,b) synthetic, (c,d) field downgoing, and (e,f) field upgoing data.

There exists a notable disparity in the amplitude ranges between synthetic (Fig 6a) and real seismograms (Fig 6c and 6e), which could introduce errors during the estimation of Thomsen’s parameters from the field dataset. Consequently, we suggest normalizing both synthetic and real seismograms by dividing the displacement of each shot gather by the largest amplitude within the gather. This normalization process condenses the intervals of both synthetic and real signals to the range [−1, 1].

To leverage the direct waves propagating in the target zone, we integrated the recorded data from the 33^rd^ to the 49^th^ receiver. The utilization of direct waves is substantiated by their high signal-to-noise (S/N) ratio, facilitating the accurate extraction of valuable features for the investigation of formations’ anisotropy. Following the identification of direct waves based on their largest amplitudes, their troughs and peaks were meticulously picked and employed as input features (Fig 6b, 6d and 6f).

The amplitude of the synthetic dataset shows a steady decrease from the 33^rd^ receiver to the 49^th^ receiver (S1 Table and S1 Fig). The peaks and troughs used as features are in the ranges 0.684635–1 and -0.227933 –-0.1496, respectively, with the highest absolute value magnitudes at the shallowest receiver numbered 33. An amplitude decrease can be noticed in the real data as well (S2 Table and S1 Fig). The peaks and troughs selected as features are in the ranges 0.51931–1 and -0.6406248 –-0.278036, respectively. The decrease can be explained by the attenuation phenomenon, namely, geometrical spreading. It is worth noticing that the medium is set elastic, meaning no absorption effect.

The 22^nd^ to 33^rd^ receivers, spanning from the top of Formation 8 to the top of Formation 10, recorded reflected waves at the interface separating Formation 10 and the reservoir zones, exhibiting a high signal-to-noise (S/N) ratio. These reflected waves encapsulate anisotropic information from both Formation 10 and the reservoir zones. The reflection coefficient is influenced by velocity contrast, which, in turn, depends on anisotropy. Identification of reflected waves was accomplished by comparing their travel times to those approximately calculated using sonic logs and trigonometric relationships to estimate ray lengths. Subsequently, the first peak and trough of the identified reflected waves were employed as features for machine learning models (Fig 6b).

The signal amplitudes of the synthetic dataset show a gradually increasing trend from the 22^nd^ receiver to the 33^rd^ receiver. The features selected from peaks and troughs are in the range 0.664336 to 1 and -0.34591 to -0.47269, respectively, as shown in S3 Table and S2 Fig. Especially the peak value from the 33^rd^ receiver is the largest among the features with 1. The features from the real dataset also display an increasing trend, even though with a slight fluctuation, as shown in S4 Table and S2 Fig. The features selected from peaks and troughs are in the range 0.413421 to 1 and -0.295633 to -1. Overall, the feature range of peak values are much closer than that of trough values.

#### 4.2.2 Frequency domain features

**Fig 7:**
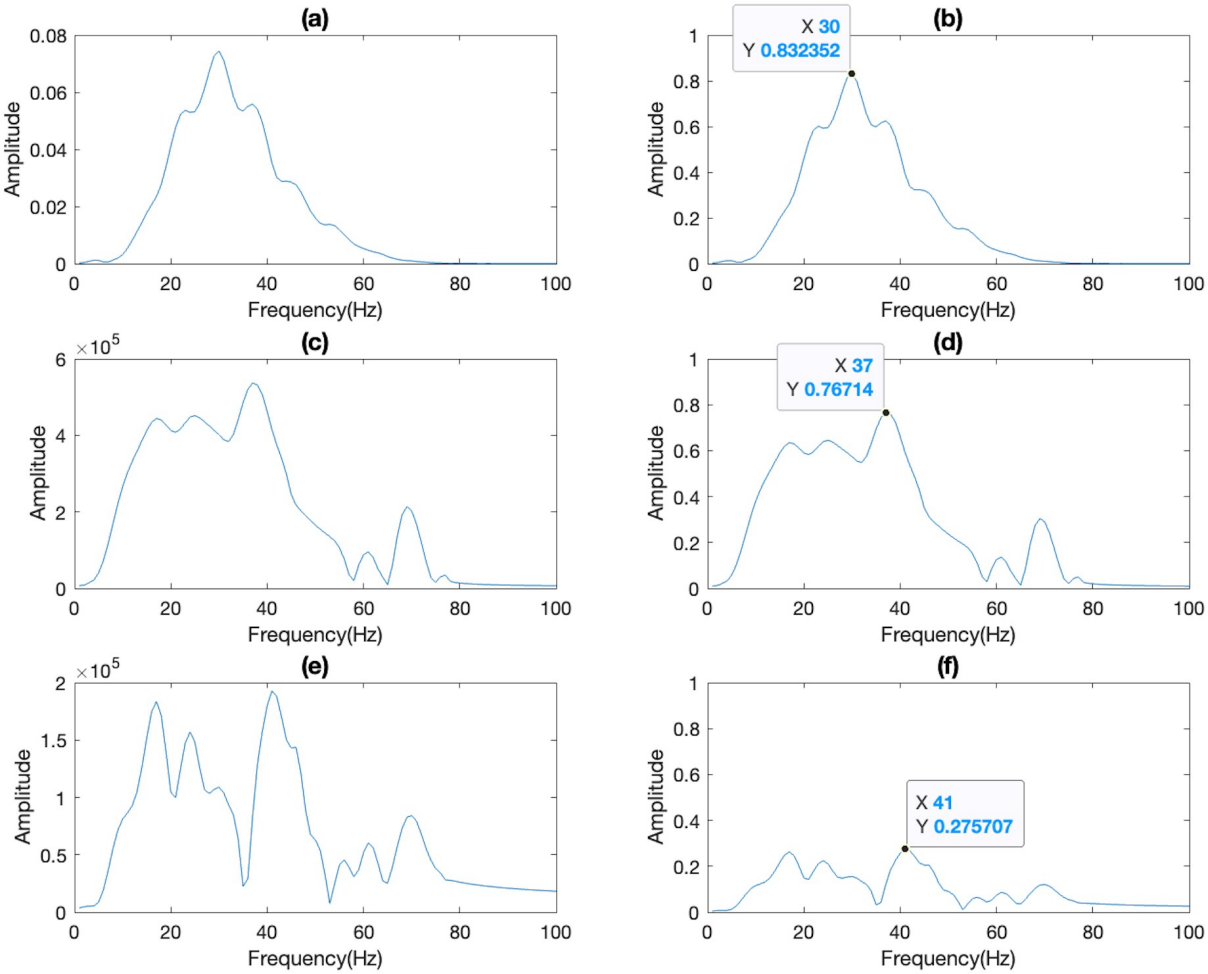
Spectra before (first column) and after (second column) normalization of (a,b) synthetic, field (c,d) downgoing, and (e,f) field upgoing data.

The Signals recorded from the 22^nd^ to the 49^th^ receivers in the time domain were transformed into the frequency domain using Fast Fourier Transform (FFT). The goal of this transformation is to identify potential machine learning (ML) features in the frequency domain that hold significant potential for anisotropy prediction. Similar to the time domain, a noticeable difference between synthetic and real spectra is apparent (Fig 7a, 7c and 7e). To address this difference, the spectra were normalized by dividing them by the maximum amplitude in each shot gather (Fig 7b, 7d and 7f), resulting in amplitudes spanning the range [0, 1]. The peak amplitudes of synthetic spectra were then selected as ML features for anisotropy prediction (Fig 7b).

In terms of the synthetic dataset, the frequency features selected from peaks range from 0.671031 to 1, as shown in S5 Table and S3 Fig. The differences between minimum value and maximum value of all features are incredibly smaller than that of time features. The features from the real dataset also displayed a larger gap between minimum and maximum values compared to features of the synthetic dataset. The features are in the range from 0.506349 to 1 as shown in S6 Table and S3 Fig.

### 4.3 Hyperparameters settings of ML models

The hyperparameters are adjustable and are defined before the training phase of a machine learning model. These parameters play a crucial role in influencing the behavior and performance of the model during training, exerting a significant impact on its ability to learn and accurately predict anisotropy. Therefore, to enhance the performance of machine learning models, a process of hyperparameter optimization was conducted. This process entailed comparing the performance of trained machine learning methods across various hyperparameter values.

The SVR function was imported from the Sklearn [50] ensemble Python library to train the data with optimized soft margin hyperparameter *C* = 1000 and radial basis function (RBF) kernel with parameter $\overset{˘}{\gamma }=0.2$. The XGB function was imported from the xgboost Python package, with optimal hyperparameters n_estimators *M* = 100, learning rate *η* = 0.3, and max_depth = 6. The MLP Regression function was imported from the Scikit-learn package [50]. The MLP optimal model consists of one input layer, five hidden layers, and an output layer. The numbers of neurons in each hidden layer are 64, 256, 64, 32, and 8, respectively. Rectified linear unit activation function (ReLU) and adaptive moment estimation (Adam) optimizer were implemented. The Python’s Conv1D function layer were applied with a kernel size of 2 and output channel of 2, while two fully connected layers were defined with thirty and one node, respectively. The output of each fully connected layer is activated by the “Rectified Linear Unit” (ReLU) function. The model computes the loss function with mean squared error (MSE) and adaptive moment estimation (Adam) solver is implemented. The parameter of the epochs is set up to 1000, so that the model can undergo training set 1000 times for an accurate training and prediction.

### 4.4 Data processing

It is worth noting that employing the entire dataset for training machine learning models can result in an overfitting problem. In such cases, the model may perform exceptionally well on the training dataset but show poor performance on new dataset. This issue is related to the influence of data splitting on the performance of machine learning models and has been investigated by some scholars, who suggested the split of 70% training and 30% testing [51, 52], especially for small data, which is our case. Therefore, the dataset was randomly divided into training and testing sets with a relative size of 7:3, meaning 70% of the data was used for training, and the remaining 30% was reserved for testing the trained model. The dataset was split by importing the train_test_split function, which shuffles the dataset using random_state = 14 before splitting the data.

The features in the training dataset were normalized by the Min_Max function to the range 0–1. After fitting the scaler to the training data, the same scaler is used to transform testing data. Data normalization can reduce the influence of outliers, thus improving the performance of machine learning models. Except for Extreme Gradient Boosting (XGB), all other ML methods require data normalization before the training and testing.


$$x_{scaled}=\frac{x-x_{min}}{x_{max}-x_{min}}$$ (14)

### 4.5 ML training results

**Fig 8:**
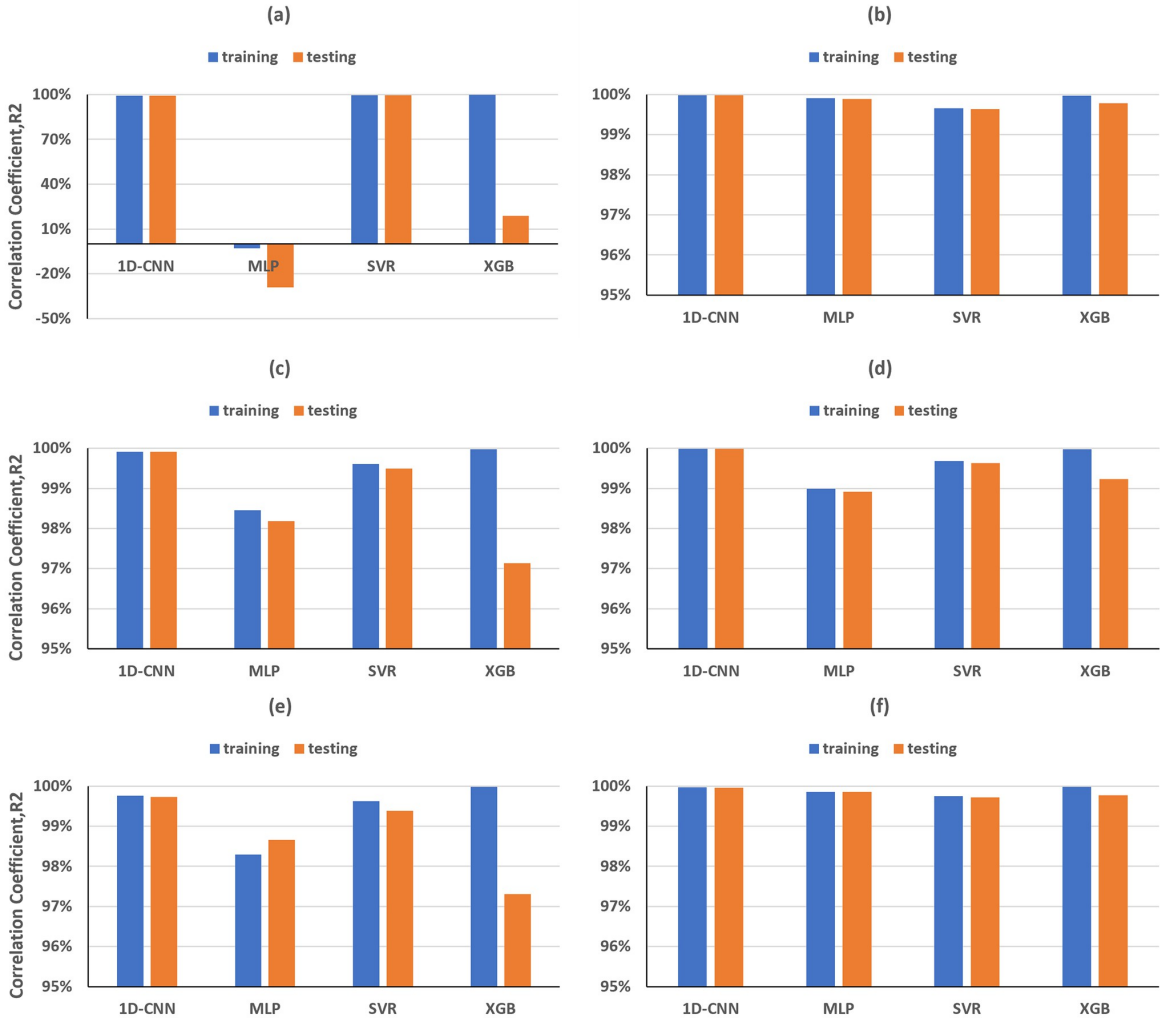
Time domain R-squared values for training and testing dataset for predicting ε (first column) and δ (second column), achieved by different MLs trained on (a, b) direct-wave features, (c, d) reflected-wave features and (e, f) combined direct and reflected features.

**Fig 9:**
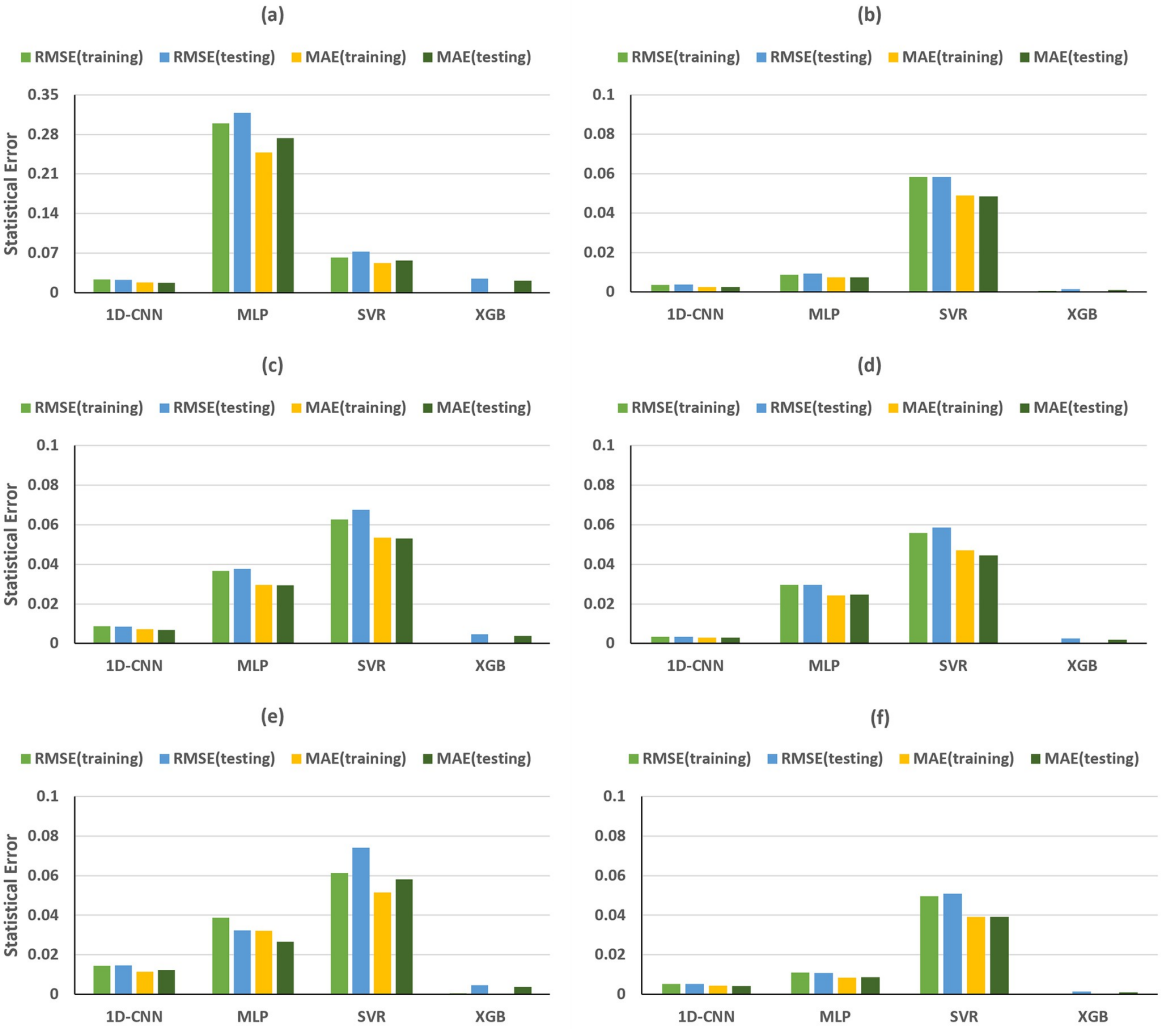
Time domain training and testing RMSE and MAE for predicting ε (first column) and δ (second column), achieved by different MLs trained on (a, b) direct-wave features, (c, d) reflected-wave features and (e, f) combined direct and reflected features.

As illustrated in Fig 8a, the training and testing of 1D-CNN and SVR models, based on the peaks and troughs of direct waves in the time domain, gave excellent R-squared values for *ε*. However, the MLP model exhibited underfitting issues, displaying negative R-squared values for training and testing dataset. Meanwhile, the RMSE and MAE values are the largest among the four machine learning methods (Fig 9a). In the case of XGB models, an overfitting problem emerged, with R-squared values for training and testing dataset reaching nearly 100% and 16%, respectively (Fig 8a). At the same time, the RMSE and MAE for testing dataset are obviously larger than those for training dataset (Fig 9a). Nevertheless, all machine learning models performed exceptionally well in predicting *δ*, achieving R-squared values for training and testing dataset exceeding 99%, with RMSE and MAE close to 0 (Figs 8b and 9b).

The performance of machine learning models based on reflected wave features is overall superior to that of direct waves for *ε* prediction (Fig 8c). Notably, the previous underfitting issue of the MLP model has been resolved, with R-squared values for the training and testing dataset reaching 98.77% and 98.30%, respectively. Similarly, in the case of XGB, the earlier overfitting problem has been rectified, achieving R-squared values for a testing dataset of 95.71%. The outstanding performance of the 1D-CNN and SVR models for ε prediction is further confirmed with both models achieving R-squared values for training and testing dataset exceeding 99%, RMSE and MAE below 0.02 (Figs 8c and 9c), using reflected wave features.

Ultimately, both direct-wave and reflected-wave features were combined in the ML models utilized to predict Thomsen’s parameters. In Figs 8e, 8f, 9e and 9f, it is evident that the majority of trained ML models demonstrated similar R-squared values and statistical errors in both the training and testing dataset compared to models utilizing single reflected-wave features.

**Fig 10:**
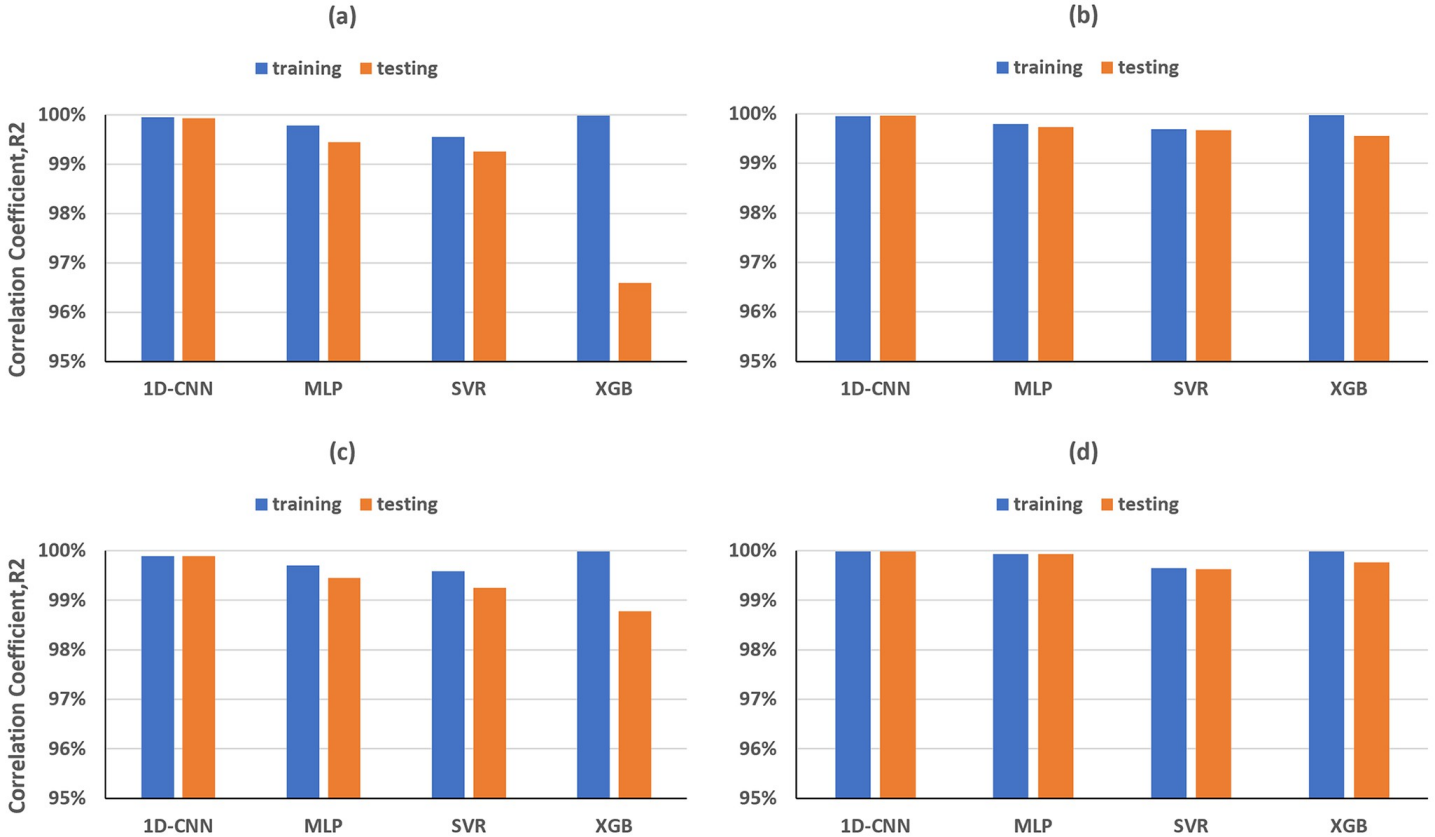
Time domain R-squared values for training and testing dataset for predicting (a, c) *ε* and (b, d) *δ*, achieved by different MLs trained on the frequency domain (upper row) features and on the combination with time domain features (lower row).

**Fig 11:**
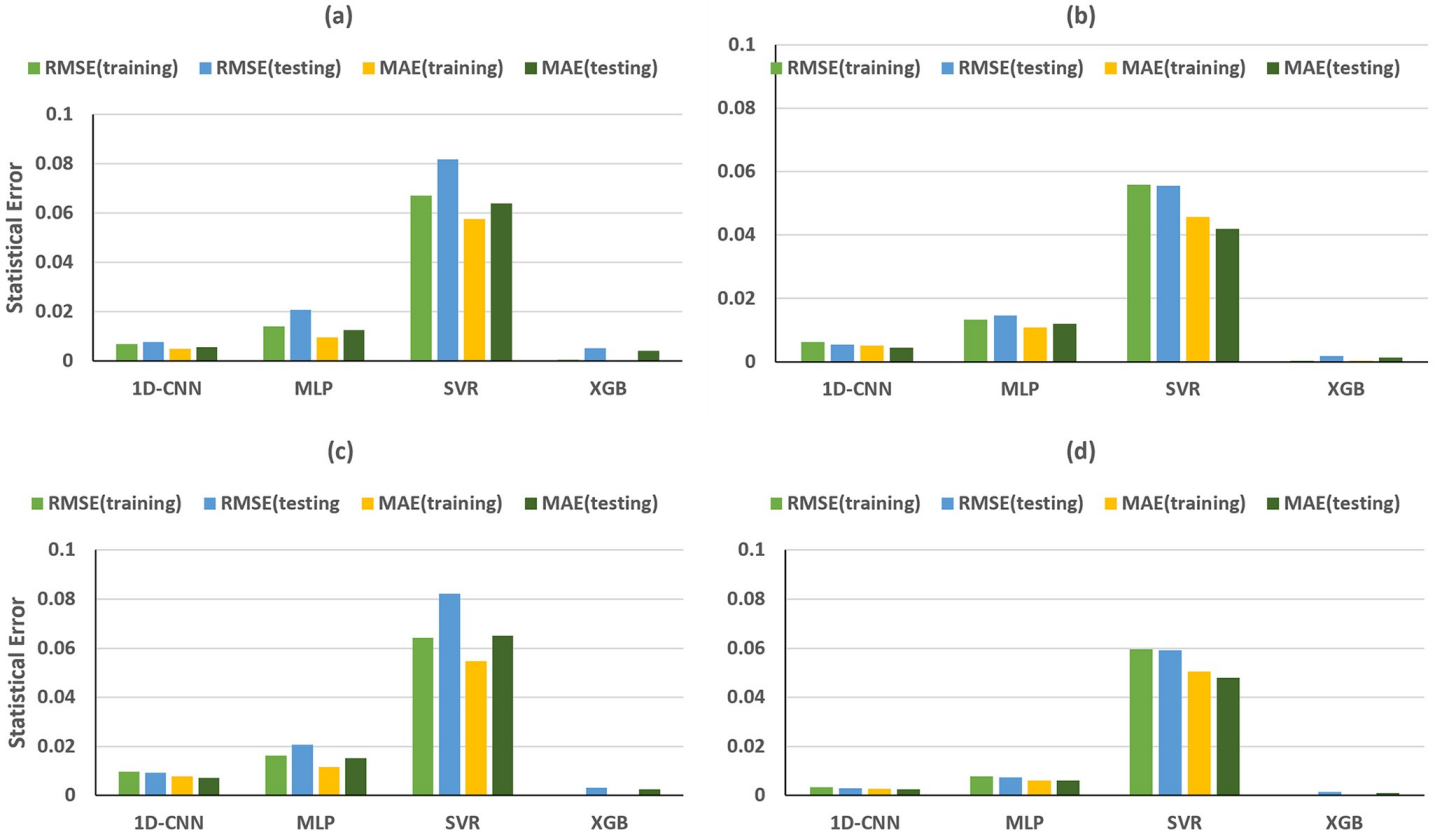
Training and testing RMSE and MAE for predicting (a, c) *ε* and (b, d) *δ*, achieved by different MLs trained on the frequency domain (upper row) features and on the combination with time domain features (lower row).

The ML models employing the peak of the dataset spectra as a feature exhibited excellent performance, achieving R-squared values for training and testing dataset over 99% for predicting both ε and δ, except for the XGB model in predicting ε, which still showed a slight overfitting issue (Fig 10a and 10b). Moreover, while estimating ε, RMSE and MAE product by SVR decreased by 0.02 compared to time domain features (Fig 11a).

After testing the performances of time domain and frequency features on the ML training, we finally investigated their combination on the prediction of Thomsen’s parameters. Compared to the above results, the performances of all ML models have been slightly improved for the prediction of both *ε* and *δ* parameters. Furthermore, the use of combined time and frequency domain features solved the overfitting issue in XGB model, which can be seen in the prediction of *ε* (Fig 10c), where the training and testing have been achieved with R-squared of 99.98% and 98.77%, respectively.

**Fig 12:**
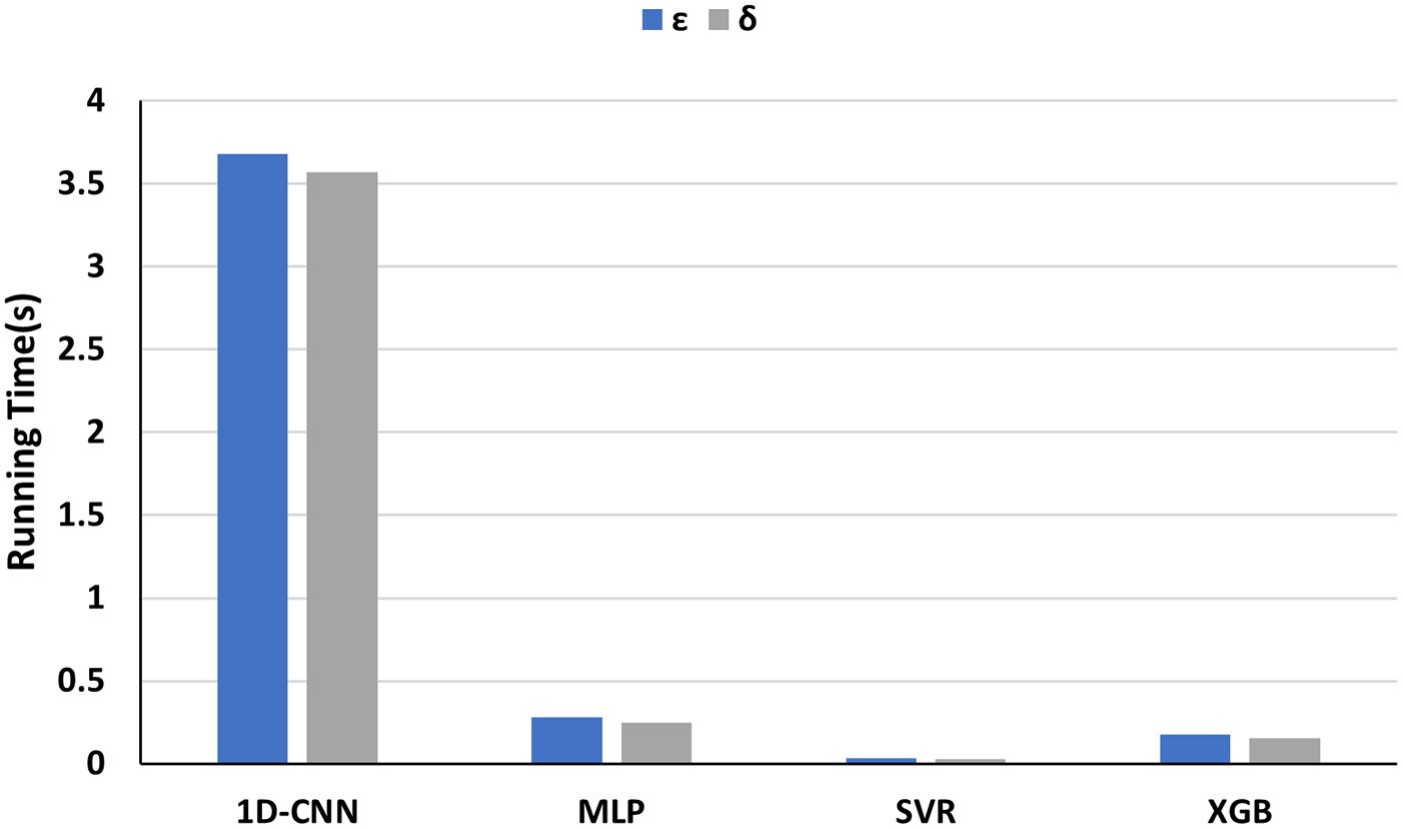
Running time for training machine learning models to *ε* and *δ*.

Meanwhile, we compared the running time of each machine learning method for training the model on the combined-features dataset (Fig 12). SVR took the least time to train the model with less than 0.1s. By contrast, 1D-CNN spends the highest running time for training, exceeding 3.5s. Furthermore, the training time of all machine learning methods for *ε* prediction is always slightly higher than that on *δ*.

### 4.6 Explainable AI

Lundberg and Lee [49] proposed the SHAP framework for elucidating machine learning model predictions. Derived from cooperative game theory [53], the Shapley value represents the average marginal contribution of a feature when considering all possible coalitions of features. To compute shapley values, SHAP applies the concept of ’coalitions’ to the feature space, evaluating all possible subsets of features and measuring their impact on the model’s prediction. By considering all combinations, SHAP captures interactions and dependencies between features. The Shapley value explanation is expressed as an additive feature attribution method:

$$g\left(z^{\prime }\right)=\phi_{0}+\sum_{j=1}^{N}\phi_{j}z_{j}^{\prime }$$ (15)

where *g* stands for the explanation model, *z*′ ∈ {0, 1}^*N*^ is the coalition vector, *N* is maximum coalition size and $\phi_{j}\in \mathbb{R}$ is the feature attribution for feature *j*, the Shapley value.

**Fig 13:**
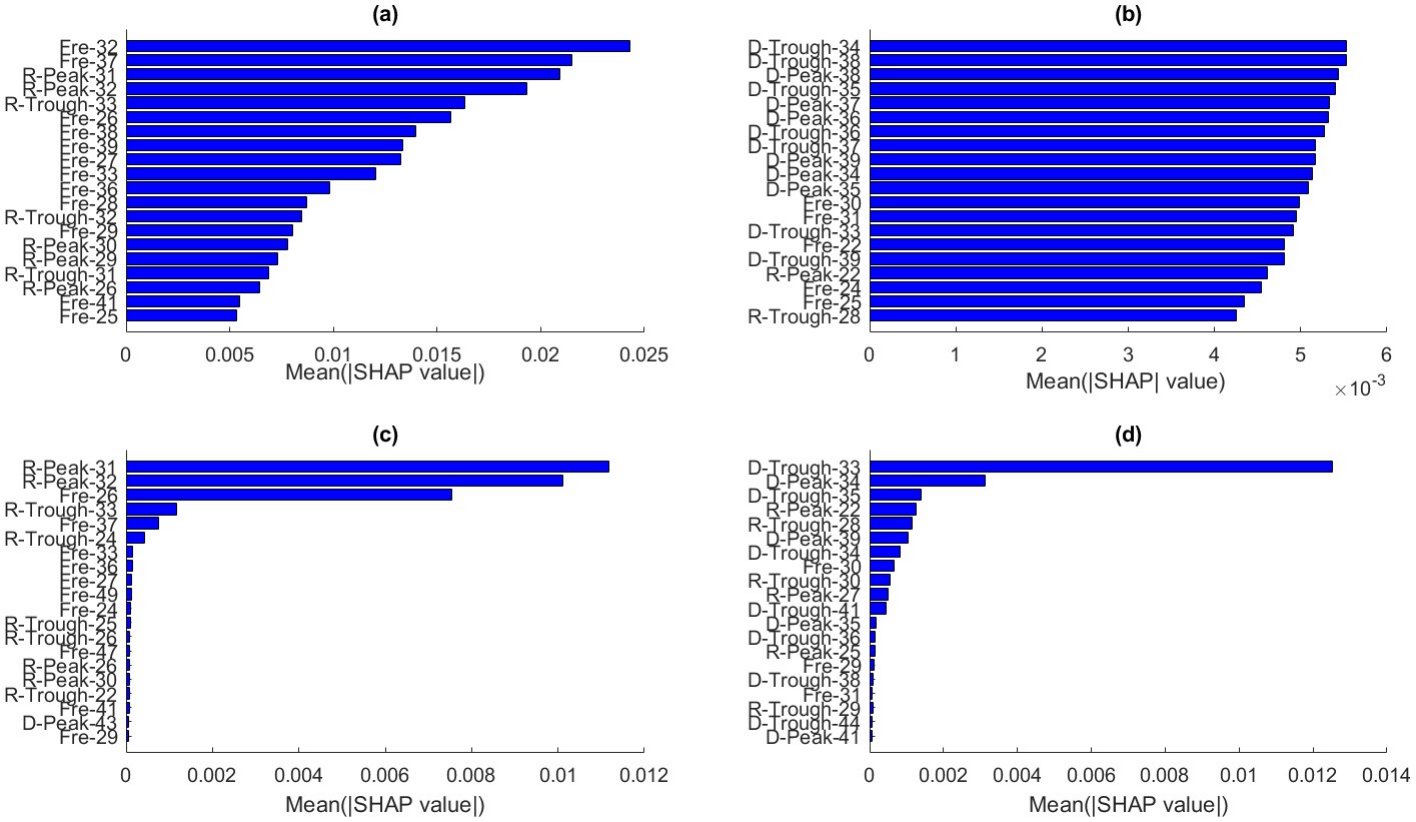
Average values of absolute SHAP values of each feature the prediction for *ε* (first column) and *δ* (second column), achieved by (a, d) SVR (a) and (c, d) XGB.

**Fig 14:**
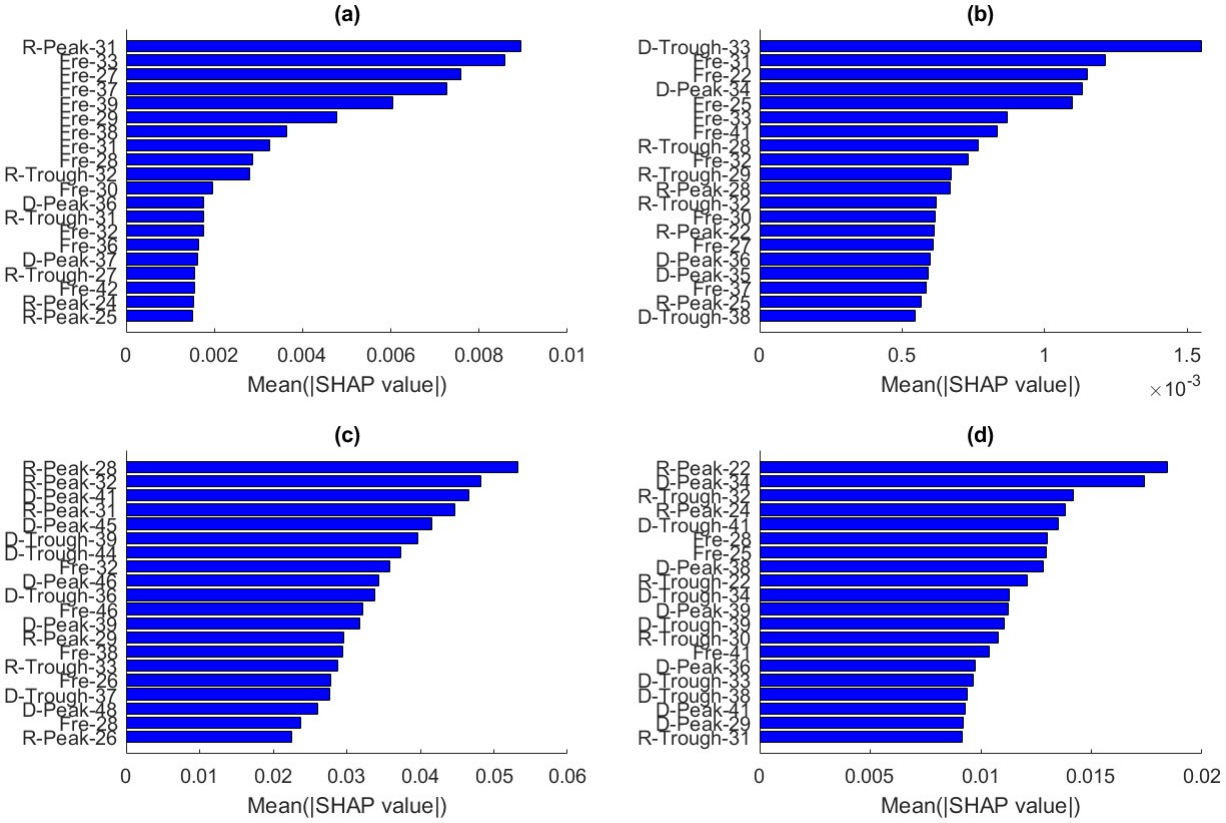
Average values of absolute SHAP values of each feature on the prediction for *ε* (first column) and *δ* (second column), achieved by (a, b) 1D-CNN and (c, d) MLP.

The SHAP explanation model was employed to elucidate the importance of each feature in ML models trained on combined time and frequency domain features. Input feature names are displayed on the y-axis, while their corresponding impacts (SHAP values) on the output are shown on the x-axis (Figs 13 and 14). Time domain features are denoted by two symbols followed by the receiver number: ’D’ or ’R’ designates features from direct and reflected waves, respectively, and ’Peak’ or ’Trough’ indicates whether the feature is extracted from the peak or trough of the waveforms. Frequency domain features are represented by ’Fre’ followed by the receiver number. For example, ’R-Peak-31’ is the peak of the reflected wave recorded by receiver number 31, while ’Fre-31’ is the peak of the spectrum of the wave recorded by the 31^st^ receiver.

SVR and XGB methods exhibit similar variation of SHAP values with features for predicting ε and δ. The SHAP values of SVR and XGB indicate that most input features affecting *ε* prediction are frequency domain and reflected-wave features (Fig 13a and 13c). In contrast, direct-wave features play a more significant role in predicting δ (Fig 13b and 13d).

While both 1D-CNN and MLP methods use the backpropagation approach to update model weights, they display different SHAP value distributions on the output. 1D-CNN results show that frequency domain features are crucial for predicting ε, with reflected-wave features having more impact on ε than direct-wave features (Fig 14a). For predicting δ, SHAP values of time and frequency domain features are closely aligned (Fig 14b). In the MLP model, time domain features play a more substantial role in predicting both ε and δ, as depicted in Fig 14c and 14d. Additionally, the impacts of direct-wave and reflected-wave features on the output are very close to each other.

## 5. ML prediction of Thomson’s parameters from field dataset

### 5.1 Near offset shot gather

After successfully training the ML models, we applied field data to these trained models to predict the values of ε and δ for our target formation, number 10, using field Vertical Seismic Profiling (VSP) data. Similar to the synthetic data case, we used the trough and peak from downgoing and upgoing waves in the time domain (Fig 6d and 6f) as input features for ML prediction. Additionally, the peak of downgoing waves’ spectra was employed as frequency domain features for ML model predictions (Fig 7d).

The ML features were extracted from waveforms recorded by twenty-five sets of shot gathers, recorded at different azimuths, and generated at an offset of 130 m consistent with the offset used previously to generate synthetic seismograms. Thus, each ML model predicts ε and δ at each shot gather. The average of the predicted values of ε and δ in Formation 10 was then compared to the inverted ones (Fig 3c), which were used as a reference for evaluating the ML prediction accuracy.

The ML models utilized combined time domain features extracted from direct and reflected waves, frequency domain features, and combined time and frequency domain features. The difference between the inverted and the predicted ε and δ in Formation 10 was quantified by the relative errors *ε*_*err*_ and *δ*_*err*_, calculated as follows:

$$\varepsilon_{err}=\frac{\varepsilon_{inverted}-\varepsilon_{ML}}{\varepsilon_{inverted}},\;\;\delta_{err}=\frac{\delta_{inverted}-\delta_{ML}}{\delta_{inverted}}$$ (16)

Thomson’s parameters ε and δ, predicted through the inversion method and the fourth trained ML methods utilizing different features, are presented in Table 2. Among these methods, 1D-CNN and SVR exhibited the closest similarity to ML-inversion, with *ε*_*err*_ and *δ*_*err*_ of 10.52% and 19.04%, respectively. On the contrary, MLP and XGB performances displayed the greatest discrepancy, reflected in the largest *ε*_*err*_ and *δ*_*err*_.

**Table 2:** Predicted ε and δ based on combined direct and reflection time domain features.

Method	*ε*		δ	
	Predicted value	Relative Error (*ε*_*err*_)	Predicted Value	Relative Error (*δ*_*err*_)
Slowness Polarization Inversion	0.171		-0.042	
1D-CNN	0.153	10.52%	-0.053	26.19%
MLP	0.111	35.08%	-0.024	42.85%
SVR	0.146	14.61%	-0.050	19.04%
XGB	0.119	30.40%	-0.023	45.23%

The ML-based prediction of ε and δ, achieved through training based on frequency domain features, is presented in Table 3. Notably, the performances of all ML models have significantly improved compared to those trained on time domain features (Table 2). The relative errors, *ε*_*err*_ and *δ*_*err*_, span in the ranges of 2.92–24.56% and 7.14–14.28%, respectively.

**Table 3:** Predicted ε and δ based on frequency domain features.

Method	*ε*		δ	
	Predicted value	Relative Error(*ε*_*err*_)	Predicted Value	Relative Error (*δ*_*err*_)
Slowness Polarization Inversion	0.171		-0.042	
1D-CNN	0.176	2.92%	-0.048	14.28%
MLP	0.158	7.60%	-0.047	11.90%
SVR	0.150	12.28%	-0.045	7.14%
XGB	0.129	24.56%	-0.045	7.14%

Table 4 displays the prediction of ε and δ based on ML models trained on combined time and frequency domain features. The 1D-CNN and SVR methods exhibit the smallest relative errors compared to the inverted ε and δ, with *ε*_*err*_ and *δ*_*err*_ of 5.26% and 11.90%, respectively. In contrast, the XGB method shows the lowest similarities to the inverted ε and δ, with *ε*_*err*_ and *δ*_*err*_ values of 35.09% and 28.57%, respectively.

**Table 4:** Predicted ε and δ based on combined time and frequency domain features.

Method	ε		δ	
	Predicted value	Relative Error (*ε*_*err*_)	Predicted Value	Relative Error (*δ*_*err*_)
Slowness Polarization Inversion	0.171		-0.042	
1D-CNN	0.162	5.26%	-0.049	16.67%
MLP	0.150	12.28%	-0.047	11.90%
SVR	0.148	13.45%	-0.048	14.29%
XGB	0.111	35.09%	-0.030	28.57%

### 5.2 Multi-offset shot gathers

Based on the results presented above, the most accurate ML-based predictions of ε and δ were achieved with frequency domain features (Table 3). Consequently, we proceeded to extract frequency domain features from VSP shot gathers recorded in the offset range of 180–2000 m, spaced at 50 m intervals, and with azimuth angles of 0° and 180°. These features were then fed into the ML models to predict ε and δ, enabling the assessment of the models’ generalization ability. Given that the azimuth angles are in the same direction (0° and 180°), ML models are expected to predict similar ε and δ values at both azimuths. Thus, the degree of similarity between Thomson’s parameters estimated at the two azimuths serves as a valuable metric for evaluating the accuracy of the ML models.

**Fig 15:**
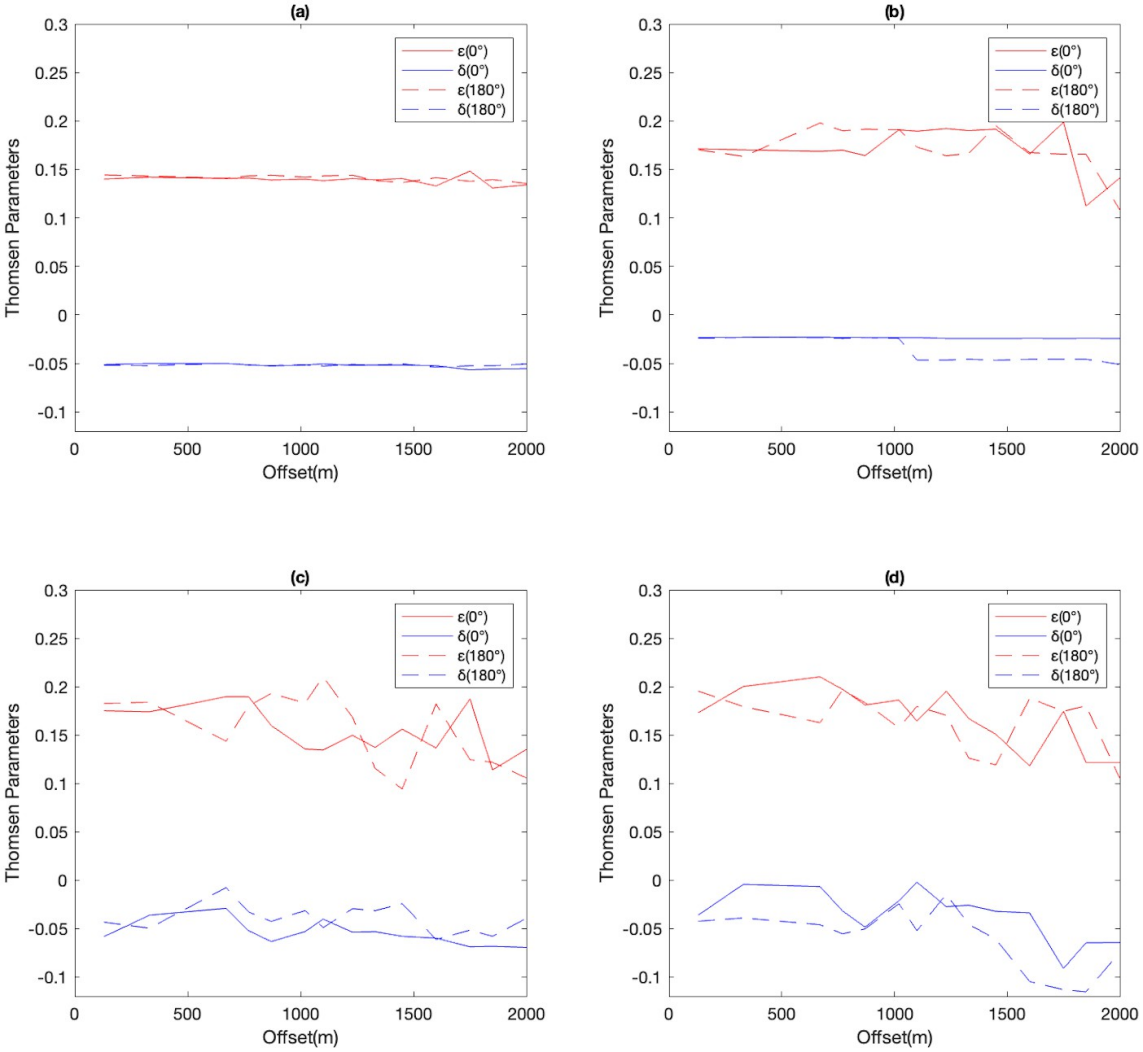
Estimated ε and δ from field data recorded at different offsets with 0° and 180° azimuths, achieved by (a) XGB, (b) SVR, (c) MLP and (d)1D-CNN.

In the estimated results for the target layer obtained by the XGB predictions (Fig 15a), ε values estimated along the 0° and 180° azimuth directions fluctuate from 180 m to 1800 m offset before experiencing a large decrease to 0.1. The difference between the predictions at the two directions is around 0.05. The parameter δ along the 0° and 180° azimuth directions remains almost constant with values around -0.025 throughout the field, showing a decrease for both directions beyond 1000 m offset.

In the case of the SVR model (Fig 15b), the predicted values of ε along the 0° and 180° azimuth directions remain stable and fluctuate around the value of 0.18 before 1600 m offset. The ε along the 0° azimuth direction displays slight fluctuations between 1600 m and 2000 m offsets, while ε along the 180° azimuth remains relatively constant. The δ values for both the 0° and 180° azimuth directions are remarkably similar, fluctuating slightly between 180 m to 1600 m. However, beyond the 1600 m offset, a small difference becomes visible between the predictions at the two directions.

Moving on to the predicted values of ε achieved by the MLP method along the 0° and 180° azimuth directions (Fig 15c), there is a decreasing trend from 0.2 to 0.1 with strong fluctuations. The largest difference between them occurs at around 1100 m offset, reaching approximately 0.08. For the parameter δ predicted by the MLP method along the 0° and 180° azimuth directions, there is weak fluctuation around -0.05, with small differences between the two directions.

Finally, the 1D-CNN predictions (Fig 15d), show that ε values along the 0° and 180° azimuth directions exhibit a decrease from around 0.2 to 0.1 with strong fluctuations. The difference between them remains below 0.05. Similarly, δ values along the 0° and 180° azimuth directions display strong fluctuations around the value of -0.05, with the difference between them also staying below 0.05.

## 6. Discussion

The ML models trained on combined time and frequency domain features showed the best performance on the synthetic dataset. This may suggest that the increase in the size of input features and their diversity has a positive impact on the performance of ML methods for predicting Thomson’s parameters. Furthermore, the accuracy of ML methods improves when they are trained on reflected-wave features rather than direct-wave features, indicating that reflected-wave features provide more accurate anisotropy information than direct-wave features. In terms of a comparison between ML methods, the 1D-CNN method achieved the highest training and testing accuracies for all features, whereas the XGB model achieved the lowest accuracy among the four ML models. It is also worth noting that the performances of all ML models are better when predicting δ than ε, suggesting that our selected features are more sensitive to the prediction of δ than ε. The impact of features on the prediction of Thomson’s parameters depends on the chosen ML method. For instance, frequency domain features have a higher impact than time domain features on ε prediction when training 1D-CNN, XGB, and SVR methods. Conversely, the inverse was noticed when training the MLP method. Furthermore, time domain features have a higher impact than frequency features on δ prediction when training MLP, XGB, and SVR models, while the impact is similar in the case of the 1D-CNN model.

**Fig 16:**
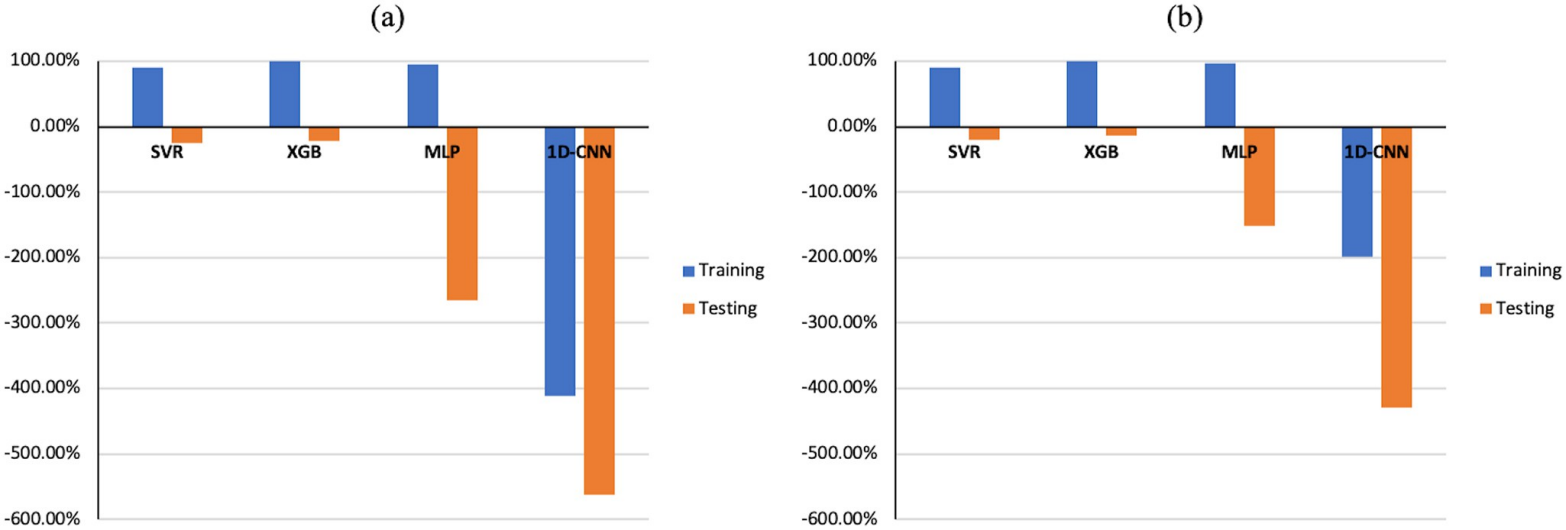
Training and testing accuracy for predicting (a) *ε* and (b) *δ*, achieved by different MLs trained on the combination of time domain features, after adding the maximum 5% random noise.

The difference between the ML predictions of ε and δ in shale formation number 10, and determined by physics-based inversion models, becomes more pronounced when ML methods are trained on time domain features. This discrepancy may be attributed to a significant difference in the amplitude of synthetic and real seismograms, despite the application of normalization. The difference decreases when ML models are trained on frequency domain features, which could be explained by the fact that these features exhibit less disparity with real data compared to time domain features. We propose that the disparity between synthetic and real data amplitudes primarily stems from neglecting the seismic attenuation effect in the forward modelling used to generate synthetic seismograms. Seismic attenuation is commonly divided into two components: scattering and intrinsic attenuation. Research studies in this area often report significant attenuation magnitudes due to the high heterogeneity of carbonates leading to significant scattering losses, especially in saturated zones such as reservoirs, which contribute to high intrinsic attenuation. Hence, it is recommended to consider the attenuation effect in the forward modelling employed to generate synthetic seismograms. Nevertheless, the overall results of this study are satisfactory and can be generalized to different offsets. Avoiding errors in estimating ε and δ is challenging due to various factors beyond attenuation, such as noise effects, discrepancies in acquisition geometry, and acquisition parameters. For instance, we tested the noise effect on model training by adding a maximum of 5% random noise to the original synthetic time signals. Additionally, the same method used for clean data was applied for feature selection. Consequently, XGB, SVR, and MLP exhibited serious overfitting issues, while 1D-CNN faced underfitting issues (Fig 16).

Overall, we have succeeded to estimate seismic anisotropy parameters of the shaley Formation. However, the application of the anisotropy prediction workflow to other geographical areas will be considered in the future to assess its generalization ability. Incorporating attenuation into the synthetic background model should also be considered in future, in order to make the synthetic time signal more representative of field data.

## 7. Conclusions

The ML models were trained on different combinations of features, in time and frequency domain of direct and reflected waves, leading to the below main outcomes,

- 1D-CNN achieved the best performance both in training and testing dataset, by contrast, XGB suffered the worst performance.
- The training and testing accuracies of *δ* prediction are always higher than *ε* for all ML methods.
- For most selected ML methods, frequency domain features have more impact on the prediction of *ε*, while time domain features have more impact on the *δ* prediction.
- Comparing the predicted results from the field dataset, MLs trained on the frequency domain features achieved the best performance, both for *ε* and *δ*. By contrast, the performances of MLs trained on the time domain features are the most unsatisfactory.

## Supporting information

S1 Fig The absolute value of (a) through and (b) peak amplitudes for real (red) and synthetic (black) direct waves. (TIF)

S2 Fig The absolute value of (a) through and (b) peak amplitudes for real (red) and synthetic (black) reflected waves. (TIF)

S3 Fig Peak frequencies for real (red) and synthetic (black) dataset. (TIF)

S1 Table Statistical parameters of synthetic direct-wave amplitudes used as features in ML models. The number in the feature name indicates the receiver number, which increases in depth.
(DOCX)

S2 Table Statistical parameters of real direct-wave amplitudes used as features in ML models. The number in the feature name indicates the receiver number, which increases in depth.
(DOCX)

S3 Table Statistical parameters of reflected-wave features in synthetic dataset. (DOCX)

S4 Table Statistical parameters of reflected-wave features in real dataset. (DOCX)

S5 Table Statistical parameters of frequency features in synthetic dataset. (DOCX)

S6 Table Statistical parameters of frequency features in real dataset. (DOCX)
