# Supplementary material for: Seismic anisotropy prediction using ML methods: A case study on an offshore carbonate oilfield
Source: PLoS One. 2025 Jan 7;20(1):e0311561. doi: 10.1371/journal.pone.0311561 (PMC11706415; doi:10.1371/journal.pone.0311561)
Supplement: S1 Table — The number in the feature name indicates the receiver number, which increases in depth. (DOCX) [file pone.0311561.s004.docx]

**Table S1.** Statistical parameters of synthetic direct-wave amplitudes used as features in ML models. The number in the feature name indicates the receiver number, which increases with depth.

| Feature | Average | Standard  Deviation | Min | Max |
| --- | --- | --- | --- | --- |
| D_Trough_33 | -0.2278707 | 3.7838E-05 | -0.2279333 | -0.2278022 |
| D_Peak_33 | 1 | 0 | 1 | 1 |
| D_Trough_34 | -0.2237613 | 0.00011805 | -0.2239755 | -0.2235683 |
| D_Peak_34 | 0.98570625 | 0.00054884 | 0.98480518 | 0.98668684 |
| D_Trough_35 | -0.2216628 | 0.00030006 | -0.2222042 | -0.2211674 |
| D_Peak_35 | 0.97979043 | 0.00125312 | 0.97771754 | 0.98201653 |
| D_Trough_36 | -0.2181691 | 0.00048346 | -0.2190481 | -0.2173849 |
| D_Peak_36 | 0.97016374 | 0.00200396 | 0.96684603 | 0.97372138 |
| D_Trough_37 | -0.215272 | 0.00064306 | -0.2164288 | -0.2142058 |
| D_Peak_37 | 0.98057245 | 0.00279924 | 0.97597377 | 0.98555464 |
| D_Trough_38 | -0.1880348 | 0.0007648 | -0.1894159 | -0.1867764 |
| D_Peak_38 | 0.83807112 | 0.00285513 | 0.83336462 | 0.84315785 |
| D_Trough_39 | -0.1846819 | 0.00084087 | -0.1862017 | -0.1832766 |
| D_Peak_39 | 0.82437775 | 0.00309817 | 0.81917572 | 0.82997404 |
| D_Trough_41 | -0.1764388 | 0.00081563 | -0.177987 | -0.1749521 |
| D_Peak_41 | 0.79327204 | 0.00283066 | 0.78787502 | 0.79889976 |
| D_Trough_42 | -0.1726261 | 0.0007658 | -0.1741185 | -0.171173 |
| D_Peak_42 | 0.77731522 | 0.00262186 | 0.77204312 | 0.78282287 |
| D_Trough_43 | -0.168751 | 0.00077545 | -0.1703002 | -0.167237 |
| D_Peak_43 | 0.76543358 | 0.00273286 | 0.75979749 | 0.77121761 |
| D_Trough_44 | -0.1653158 | 0.00074082 | -0.1668245 | -0.1638378 |
| D_Peak_44 | 0.75146323 | 0.00262016 | 0.74590211 | 0.75711024 |
| D_Trough_45 | -0.1615004 | 0.00076114 | -0.163096 | -0.1599632 |
| D_Peak_45 | 0.7394271 | 0.00277422 | 0.7334866 | 0.74544014 |
| D_Trough_46 | -0.1584045 | 0.00073799 | -0.1599983 | -0.1569018 |
| D_Peak_46 | 0.72684502 | 0.00269392 | 0.72097997 | 0.73272563 |
| D_Trough_47 | -0.1547395 | 0.00071805 | -0.1563384 | -0.1532729 |
| D_Peak_47 | 0.71455952 | 0.00286814 | 0.70831088 | 0.72080903 |
| D_Trough_48 | -0.1518513 | 0.00074942 | -0.1535324 | -0.1503225 |
| D_Peak_48 | 0.7033329 | 0.00280681 | 0.69716327 | 0.70945374 |
| D_Trough_49 | -0.14841 | 0.00073369 | -0.1500729 | -0.1469174 |
| D_Peak_49 | 0.69119355 | 0.00299768 | 0.68463534 | 0.69770128 |
