# Supplementary material for: Seismic anisotropy prediction using ML methods: A case study on an offshore carbonate oilfield
Source: PLoS One. 2025 Jan 7;20(1):e0311561. doi: 10.1371/journal.pone.0311561 (PMC11706415; doi:10.1371/journal.pone.0311561)
Supplement: S2 Table — The number in the feature name indicates the receiver number, which increases in depth. (DOCX) [file pone.0311561.s005.docx]

**Table S2.** Statistical parameters of real direct-wave amplitudes used as features in ML models. The number in the feature name indicates the receiver number, which increases in depth.

|  | Average | Standard  Deviation | Minimum | Maximum |
| --- | --- | --- | --- | --- |
| D_Peak_33 | 0.99725556 | 0.01018692 | 0.95365244 | 1 |
| D_Trough_33 | -0.569833 | 0.03213498 | -0.6406248 | -0.5104741 |
| D_Peak_34 | 0.91540994 | 0.01558436 | 0.88990684 | 0.9572044 |
| D_Trough_34 | -0.4563144 | 0.0178569 | -0.4994801 | -0.428625 |
| D_Peak_35 | 0.83995263 | 0.01917533 | 0.79151949 | 0.86955641 |
| D_Trough_35 | -0.402317 | 0.02445273 | -0.4490681 | -0.3496736 |
| D_Peak_36 | 0.77692775 | 0.03239345 | 0.68776036 | 0.8238648 |
| D_Trough_36 | -0.3764127 | 0.02702013 | -0.4197107 | -0.3107491 |
| D_Peak_37 | 0.78249106 | 0.03630056 | 0.68944935 | 0.84667405 |
| D_Trough_37 | -0.363799 | 0.0250986 | -0.4137017 | -0.3167278 |
| D_Peak_38 | 0.82993503 | 0.03130207 | 0.77655955 | 0.88627895 |
| D_Trough_38 | -0.3891029 | 0.02842262 | -0.4456114 | -0.3477223 |
| D_Peak_39 | 0.91837657 | 0.04207399 | 0.84019217 | 1 |
| D_Trough_39 | -0.4252817 | 0.02322879 | -0.4727832 | -0.3827522 |
| D_Peak_41 | 0.91138292 | 0.03825116 | 0.83258595 | 0.97717052 |
| D_Trough_41 | -0.4815785 | 0.02967858 | -0.5305359 | -0.4078684 |
| D_Peak_42 | 0.82211695 | 0.04141965 | 0.73919225 | 0.89619222 |
| D_Trough_42 | -0.4749861 | 0.03007874 | -0.5351167 | -0.422575 |
| D_Peak_43 | 0.81841826 | 0.03626566 | 0.73538512 | 0.86618312 |
| D_Trough_43 | -0.4360906 | 0.02273733 | -0.4796945 | -0.3801466 |
| D_Peak_44 | 0.78068795 | 0.03937914 | 0.69045801 | 0.85140866 |
| D_Trough_44 | -0.3661127 | 0.02652872 | -0.4211196 | -0.321 |
| D_Peak_45 | 0.73320334 | 0.03649255 | 0.63944145 | 0.79258822 |
| D_Trough_45 | -0.3402866 | 0.02077159 | -0.3805573 | -0.3008217 |
| D_Peak_46 | 0.69308466 | 0.03822654 | 0.60706036 | 0.74752755 |
| D_Trough_46 | -0.3772536 | 0.02673376 | -0.4288321 | -0.3276663 |
| D_Peak_47 | 0.67966944 | 0.03570699 | 0.60661737 | 0.72022766 |
| D_Trough_47 | -0.35494671 | 0.02247229 | -0.30060312 | -0.392664 |
| D_Peak_48 | 0.61967648 | 0.03284754 | 0.5392887 | 0.66253225 |
| D_Trough_48 | -0.3316528 | 0.01789862 | -0.360489 | -0.2913399 |
| D_Peak_49 | 0.60507807 | 0.03295857 | 0.51930999 | 0.64519285 |
| D_Trough_49 | -0.3186755 | 0.01952497 | -0.3525599 | -0.2780358 |
