# Supplementary material for: Seismic anisotropy prediction using ML methods: A case study on an offshore carbonate oilfield
Source: PLoS One. 2025 Jan 7;20(1):e0311561. doi: 10.1371/journal.pone.0311561 (PMC11706415; doi:10.1371/journal.pone.0311561)
Supplement: S3 Table — (DOCX) [file pone.0311561.s006.docx]

**Table S3.** Statistical parameters of reflected-wave features in synthetic dataset

|  | Average | Standard  Deviation | Minimum | Maximum |
| --- | --- | --- | --- | --- |
| R_Peak_22 | 0.666486026 | 0.00105679 | 0.664336 | 0.668294 |
| R_Trough_22 | -0.34744421 | 0.000771113 | -0.34914 | -0.34591 |
| R_Peak_24 | 0.705373249 | 0.001128966 | 0.703008 | 0.707362 |
| R_Trough_24 | -0.40490265 | 0.000319471 | -0.40568 | -0.40411 |
| R_Peak_25 | 0.782861236 | 0.001551736 | 0.779742 | 0.785683 |
| R_Trough_25 | -0.42248094 | 0.001046212 | -0.42456 | -0.42033 |
| R_Peak_26 | 0.687199707 | 0.00085999 | 0.685338 | 0.688933 |
| R_Trough_26 | -0.35845793 | 0.000586411 | -0.35972 | -0.35707 |
| R_Peak_27 | 0.726006363 | 0.001448438 | 0.722938 | 0.72859 |
| R_Trough_27 | -0.41166816 | 0.001543808 | -0.41453 | -0.4086 |
| R_Peak_28 | 0.793714081 | 0.001508133 | 0.790491 | 0.796523 |
| R_Trough_28 | -0.45054135 | 0.001786602 | -0.45369 | -0.44711 |
| R_Peak_29 | 0.726921387 | 0.001107201 | 0.724461 | 0.729028 |
| R_Trough_29 | -0.35243383 | 0.00152209 | -0.35533 | -0.34952 |
| R_Peak_30 | 0.780609186 | 0.001031694 | 0.778318 | 0.782681 |
| R_Trough_30 | -0.42763022 | 0.002119353 | -0.43151 | -0.42358 |
| R_Peak_31 | 0.75304433 | 0.000310005 | 0.752293 | 0.753845 |
| R_Trough_31 | -0.4319873 | 0.001553379 | -0.43518 | -0.42892 |
| R_Peak_32 | 0.971300595 | 0.000253534 | 0.970708 | 0.971999 |
| R_Trough_32 | -0.57018904 | 0.002429572 | -0.57515 | -0.56542 |
| R_Peak_33 | 1 | 0 | 1 | 1 |
| R_Trough_33 | -0.4699167 | 0.000962276 | -0.47269 | -0.46795 |
