# Supplementary material for: Seismic anisotropy prediction using ML methods: A case study on an offshore carbonate oilfield
Source: PLoS One. 2025 Jan 7;20(1):e0311561. doi: 10.1371/journal.pone.0311561 (PMC11706415; doi:10.1371/journal.pone.0311561)
Supplement: S4 Table — (DOCX) [file pone.0311561.s007.docx]

**Table S4.** Statistical parameters of reflected-wave features in real dataset.

|  | Average | Standard  Deviation | Minimum | Maximum |
| --- | --- | --- | --- | --- |
| R_Peak_22 | 0.57664415 | 0.08934415 | 0.41342063 | 0.72653225 |
| R_Trough_22 | -0.4431288 | 0.07189782 | -0.5809789 | -0.2956333 |
| R_Peak_24 | 0.64249738 | 0.07387611 | 0.50694413 | 0.78469815 |
| R_Trough_24 | -0.4647143 | 0.07133281 | -0.6214079 | -0.3692851 |
| R_Peak_25 | 0.68099842 | 0.06458281 | 0.60060841 | 0.84371952 |
| R_Trough_25 | -0.4880889 | 0.08777162 | -0.6630585 | -0.3463061 |
| R_Peak_26 | 0.66858497 | 0.05728038 | 0.5965259 | 0.78209635 |
| R_Trough_26 | -0.5294164 | 0.08252577 | -0.6737347 | -0.3869683 |
| R_Peak_27 | 0.68952347 | 0.03589257 | 0.6106216 | 0.73919303 |
| R_Trough_27 | -0.5463657 | 0.07386006 | -0.7226532 | -0.4291765 |
| R_Peak_28 | 0.78077298 | 0.04907578 | 0.68149856 | 0.90162934 |
| R_Trough_28 | -0.6224652 | 0.05922796 | -0.7609155 | -0.5438333 |
| R_Peak_29 | 0.83769866 | 0.05067397 | 0.77417926 | 0.98248406 |
| R_Trough_29 | -0.6917326 | 0.05576701 | -0.8084995 | -0.5918143 |
| R_Peak_30 | 0.91270459 | 0.03870409 | 0.83823153 | 1 |
| R_Trough_30 | -0.7452367 | 0.07884581 | -0.8851133 | -0.6186178 |
| R_Peak_31 | 0.96993561 | 0.0321274 | 0.90398846 | 1 |
| R_Trough_31 | -0.8162519 | 0.07287367 | -0.9086429 | -0.704105 |
| R_Peak_32 | 0.93638726 | 0.053787 | 0.82741742 | 1 |
| R_Trough_32 | -0.9111015 | 0.08222239 | -1 | -0.7441066 |
| R_Peak_33 | 0.93964512 | 0.05852723 | 0.82075773 | 1 |
| R_Trough_33 | -0.9350889 | 0.06430744 | -1 | -0.8201329 |
