# Supplementary material for: Seismic anisotropy prediction using ML methods: A case study on an offshore carbonate oilfield
Source: PLoS One. 2025 Jan 7;20(1):e0311561. doi: 10.1371/journal.pone.0311561 (PMC11706415; doi:10.1371/journal.pone.0311561)
Supplement: S5 Table — (DOCX) [file pone.0311561.s008.docx]

**Table S5.** Statistical parameters of frequency features in synthetic dataset.

|  | Average | Standard  Deviation | Minimum | Maximum |
| --- | --- | --- | --- | --- |
| Fre_22 | 0.947668 | 0.000594 | 0.946575 | 0.948731 |
| Fre_24 | 0.901226 | 0.000376 | 0.900503 | 0.901938 |
| Fre_25 | 0.909049 | 0.000333 | 0.90841 | 0.909657 |
| Fre_26 | 0.828012 | 3.91E-05 | 0.82792 | 0.828107 |
| Fre_27 | 0.858929 | 0.000113 | 0.858673 | 0.859155 |
| Fre_28 | 0.892265 | 0.000382 | 0.891509 | 0.892977 |
| Fre_29 | 0.794645 | 0.000218 | 0.79417 | 0.79502 |
| Fre_30 | 0.829212 | 0.000319 | 0.828617 | 0.829729 |
| Fre_31 | 0.865529 | 0.000474 | 0.864661 | 0.866376 |
| Fre_32 | 0.896557 | 8.39E-05 | 0.896357 | 0.896805 |
| Fre_33 | 0.998024 | 0.000555 | 0.996816 | 0.999298 |
| Fre_34 | 0.870402 | 0.000184 | 0.870044 | 0.870936 |
| Fre_35 | 1 | 0 | 1 | 1 |
| Fre_36 | 0.792907 | 0.000784 | 0.791003 | 0.794608 |
| Fre_37 | 0.995161 | 0.000755 | 0.993484 | 0.996811 |
| Fre_38 | 0.770879 | 0.000721 | 0.769187 | 0.772591 |
| Fre_39 | 0.755059 | 0.000668 | 0.753344 | 0.756779 |
| Fre_41 | 0.731906 | 0.000701 | 0.730315 | 0.733504 |
| Fre_42 | 0.725574 | 0.000885 | 0.723505 | 0.727533 |
| Fre_43 | 0.719581 | 0.001131 | 0.716788 | 0.722124 |
| Fre_44 | 0.71312 | 0.001368 | 0.709941 | 0.716384 |
| Fre_45 | 0.704968 | 0.001442 | 0.701767 | 0.708435 |
| Fre_46 | 0.696217 | 0.001359 | 0.693292 | 0.699462 |
| Fre_47 | 0.689366 | 0.001355 | 0.686627 | 0.692218 |
| Fre_48 | 0.680925 | 0.001429 | 0.678124 | 0.683668 |
| Fre_49 | 0.674059 | 0.001527 | 0.671031 | 0.676968 |
