# Supplementary material for: Seismic anisotropy prediction using ML methods: A case study on an offshore carbonate oilfield
Source: PLoS One. 2025 Jan 7;20(1):e0311561. doi: 10.1371/journal.pone.0311561 (PMC11706415; doi:10.1371/journal.pone.0311561)
Supplement: S6 Table — (DOCX) [file pone.0311561.s009.docx]

**Table S6.** Statistical parameters of frequency features in real dataset.

|  | Average | Standard  Deviation | Minimum | Maximum |
| --- | --- | --- | --- | --- |
| Fre_22 | 0.687318 | 0.090169 | 0.506349 | 0.852258 |
| Fre_24 | 0.675528 | 0.101869 | 0.446795 | 0.85199 |
| Fre_25 | 0.679141 | 0.097556 | 0.51574 | 0.870298 |
| Fre_26 | 0.661429 | 0.060085 | 0.513604 | 0.766697 |
| Fre_27 | 0.763093 | 0.061249 | 0.641477 | 0.898358 |
| Fre_28 | 0.789173 | 0.051374 | 0.700603 | 0.892834 |
| Fre_29 | 0.794495 | 0.055994 | 0.686698 | 0.940557 |
| Fre_30 | 0.872363 | 0.058998 | 0.759606 | 0.963145 |
| Fre_31 | 0.901406 | 0.056321 | 0.806529 | 1 |
| Fre_32 | 0.956003 | 0.050259 | 0.819152 | 1 |
| Fre_33 | 0.986599 | 0.019429 | 0.946282 | 1 |
| Fre_34 | 0.949916 | 0.031801 | 0.885898 | 1 |
| Fre_35 | 0.829165 | 0.034853 | 0.741869 | 0.875243 |
| Fre_36 | 0.815726 | 0.049478 | 0.712603 | 0.893417 |
| Fre_37 | 0.799075 | 0.03943 | 0.726728 | 0.859504 |
| Fre_38 | 0.836019 | 0.057986 | 0.736182 | 0.956161 |
| Fre_39 | 0.910141 | 0.054755 | 0.801765 | 1 |
| Fre_41 | 0.947297 | 0.047944 | 0.840891 | 1 |
| Fre_42 | 0.866151 | 0.044381 | 0.771845 | 0.986518 |
| Fre_43 | 0.876752 | 0.048543 | 0.773797 | 0.980469 |
| Fre_44 | 0.827233 | 0.055212 | 0.717082 | 0.91735 |
| Fre_45 | 0.804857 | 0.080883 | 0.606544 | 0.911295 |
| Fre_46 | 0.730037 | 0.057954 | 0.623446 | 0.819268 |
| Fre_47 | 0.741691 | 0.078086 | 0.565616 | 0.873511 |
| Fre_48 | 0.702821 | 0.074342 | 0.534245 | 0.86503 |
| Fre_49 | 0.690481 | 0.07321 | 0.518014 | 0.850464 |
